# Identification and functional characterization of the dirigent gene family in *Phryma leptostachya* and the contribution of *PlDIR1* in lignan biosynthesis

**DOI:** 10.1186/s12870-023-04297-6

**Published:** 2023-05-31

**Authors:** Yakun Pei, Wenhan Cao, Wenwen Yu, Chaoyang Peng, Wenhao Xu, Yayun Zuo, Wenjun Wu, Zhaonong Hu

**Affiliations:** 1 Institute of Pesticide Science, College of Plant Protection, Northwest A & F University, Yangling, 712100 Shaanxi China; 2 Key Laboratory for Botanical Pesticide R & D of Shaanxi Province, Yangling, 712100 Shaanxi China; 3 Key Laboratory of Integrated Pest Management On Crops in Northwestern Loess Plateau, Ministry of Agriculture, Yangling, 712100 Shaanxi China

**Keywords:** *Phryma leptostachya*, Dirigent protein, Lignan biosynthesis, ( +)-Pinoresinol-formation protein, Catalytic activity

## Abstract

**Background:**

Furofuran lignans, the main insecticidal ingredient in *Phryma leptostachya*, exhibit excellent controlling efficacy against a variety of pests. During the biosynthesis of furofuran lignans, Dirigent proteins (DIRs) are thought to be dominant in the stereoselective coupling of coniferyl alcohol to form ( ±)-pinoresinol. There are DIR family members in almost every vascular plant, but members of DIRs in *P. leptostachya* are unknown. To identify the *PlDIR* genes and elucidate their functions in lignan biosynthesis, this study performed transcriptome-wide analysis and characterized the catalytic activity of the PlDIR1 protein.

**Results:**

Fifteen full-length unique *PlDIR* genes were identified in *P. leptostachya*. A phylogenetic analysis of the *PlDIRs* classified them into four subfamilies (DIR-a, DIR-b/d, DIR-e, and DIR-g), and 12 conserved motifs were found among them. In tissue-specific expression analysis, except for *PlDIR7*, which displayed the highest transcript abundance in seeds, the other *PlDIRs* showed preferential expression in roots, leaves, and stems. Furthermore, the treatments with signaling molecules demonstrated that *PlDIRs* could be significantly induced by methyl jasmonate (MeJA), salicylic acid (SA), and ethylene (ETH), both in the roots and leaves of *P. leptostachya*. In examining the tertiary structure of the protein and the critical amino acids, it was found that PlDIR1, one of the DIR-a subfamily members, might be involved in the region- and stereo-selectivity of the phenoxy radical. Accordingly, LC–MS/MS analysis demonstrated the catalytic activity of recombinant PlDIR1 protein from *Escherichia coli* to direct coniferyl alcohol coupling into ( +)-pinoresinol. The active sites and hydrogen bonds of the interaction between PlDIR1 and *bis*-quinone methide (bisQM), the intermediate in ( +)-pinoresinol formation, were analyzed by molecular docking. As a result, 18 active sites and 4 hydrogen bonds (Asp-42, Ala-113, Leu-138, Arg-143) were discovered in the PlDIR1-bisQM complex. Moreover, correlation analysis indicated that the expression profile of *PlDIR1* was closely connected with lignan accumulations after SA treatment.

**Conclusions:**

The results of this study will provide useful clues for uncovering *P. leptostachya*'s lignan biosynthesis pathway as well as facilitate further studies on the DIR family.

**Supplementary Information:**

The online version contains supplementary material available at 10.1186/s12870-023-04297-6.

## Background

In the Himalayas, temperate Asia, and northern East America, *Phryma leptostachya* L. is a widely distributed perennial herb with both medicinal and agricultural uses [1–3]. As a traditional natural insecticide with striking insecticidal activity, this plant has been used to repel mosquitoes and flies in East Asia [4, 5]. Previous investigations have shown that the main insecticidal active ingredients in *P. leptostachya* are furofuran lignans [1, 6, 7]. For example, haedoxan A (HA) exhibits high insecticidal effectiveness against a wide variety of pests, like *Culex pipiens pallens* [7], *Mythimna separata* [4], *Aedes albopictus*, and *Aedes aegypti* [6, 8, 9]. ( +)-Phrymarolins I and II (( +)-P-I and P-II) have the same furofuran skeleton as HA and there is considerable synergistic activity between them and HA, pyrethrin, or carbamate pesticides [6, 10]. Consequently, haedoxans and phrymarolins are likely to serve as the main insecticidal ingredients in new botanical pesticides. However, due to their extremely low contents and the difficulty of chemical synthesis [11–14], a better understanding of the biosynthetic pathways of furofuran lignans in *P. leptostachya* would be an advantage to provide a potential approach for their application.

Coniferyl alcohol, one of the monolignols generated from the phenylpropanoid pathway, is dimerized to produce furofuran lignans [15, 16]. Then, a pair of methylenedioxy bridges are formed, followed by oxidation, methylation, and acetylation [17–19]. Coniferyl alcohol is therefore the monomeric building block for furofuran lignans, which can alter their composition and types significantly. To investigate the enzyme that catalyzes coniferyl alcohol, Davin et al*.* [20] conducted groundbreaking research and found that in the presence of an oxidase (peroxidase or laccase) or electron oxidant, coniferyl alcohol molecules could be stereoselectively coupled into ( +)-pinoresinol by a catalytic enzyme, dirigent protein (DIR).

The name DIRs comes from the Latin word *dirigere*, which means to align or guide. The first DIR protein was discovered in *Forsythia intermedia* [20]. Then, ferns, gymnosperms, and angiosperms were subsequently found to contain this kind of protein [21–23]. Often, *DIR* genes come in the form of gene families, such as 25, 49, 44, 45, 29, and 19 *DIRs*, which have been found in *Arabidopsis thaliana*, *Oryza sativa*, *Linum usitatissimum*, *Medicago truncatula*, *Brassica rapa*, and *Isatis indigotica* [21, 24–28]. According to Ralph et al*.* [21], six subfamilies of DIR proteins (DIR-a, DIR-b/d, DIR-c, DIR-e, DIR-f, and DIR-g) are recognized based on the lignan spatial structures they mediate and their evolutionary relationships. The DIR-a subfamily is thought to play a role in the production of pinoresinol, whereas the roles of the other subfamily members remain unknown. As a result, DIRs that do not belong to the DIR-a subfamily are referred to as DIR-like [20, 29].

By inhibiting microbe-derived degradative enzymes and forming a barrier against microbial pathogens, lignans play significant roles in plant pathogen defense. Therefore, by regulating monolignol coupling associated with the biosynthesis of lignans, DIRs improve plant stress resistance [16, 23, 30]. Numerous biotic and abiotic stressors can activate *DIR* genes. For example, *DIR* genes in the corresponding plants can be induced by the infection of pathogens, which include *Fusarium solani* in soybean [31], *Colletotrichum gloeosporioides* in *Physcomitrella patens* [32], *Erysiphe necator* in *Vitis vinifera* [33], and *Verticillium dahlia* in cotton [34]. Also, after exposure to abiotic stresses, such as salt, drought, high/low temperature, pesticide residue, water logging, and H_2_O_2_, there is evidence of *ScDIR* in sugarcane [35], *OsDIRs* and *ShDJ* in rice [36, 37], *BrDIRs* in Brassica [28], *BhDIR1* in *Boea hygrometrica* [38], and *CsDIR16* in cucumber [39] responding to them. In addition, *DIR* genes can be modulated by hormone signals, such as salicylic acid (SA), ethylene (ETH), methyl jasmonate (MeJA), and abscisic acid (ABA) [40].

*DIR* genes participate in many physiological processes in plants, and the exploration of their function is helpful to analyze lignan biosynthesis and metabolic pathways. Due to there being no detailed study of the *DIR* gene family in *P. leptostachya*, our work aims to further broaden current knowledge of the functions of *PlDIRs*. Here, a transcriptome-wide analysis of the DIR family in *P. leptostachy* was performed, and sequence characterization, phylogenetics, motif, and tertiary structure analysis were included. Meanwhile, we also investigated the expression patterns of *PlDIRs* in different tissues and explored their responses to signaling molecules. Furthermore, the function of PlDIR1 as a ( +)-pinoresinol-formation protein was revealed by analyzing the catalytic activity of its recombinant protein and the results of molecular docking. These discoveries will help comprehend *PlDIRs’* function and will establish the groundwork for understanding the biosynthetic pathways for furofuran lignans and metabolic engineering in *P. leptostachya*.

## Results

### Identification and sequence analysis of *DIR* genes in *P. leptostachya*

The members of *the P. leptostachya DIR* gene family were identified by screening the transcriptome sequencing of *P. leptostachya* (accession no. PRJNA551634). After rejecting the redundant, overlapped, incomplete, and repeated sequences, 15 *DIR* gene sequences with complete open reading frames (ORFs) were obtained and named *PlDIR1*-*15*. Their conserved DIR domains (PF03018) were analyzed with the Pfam (http://pfam.xfam.org/search) and SMART (http://smart.embl-heidelberg.de/) programs. The analysis results for these genes are shown in Table 1. It was found that the predicted ORFs for the 15 *DIR* genes ranged from 543 (*PlDIR13*) to 609 (*PlDIR4*) bp, with the amino acid length mainly between 181–203 aa. The molecular weight (MW) of PlDIRs was between 19.78–22.16 kDa. The predicted isoelectric point (pI) values were within the large variable range (4.43–10.13), and the pI of 8 members is alkaline (pI > 7.0). Furthermore, except for PlDIR9, 13 and 15, most of the PlDIRs had a 20–30 aa length signal peptide at the *N*-terminus.

**Table 1:** Sequence analysis of 15 *DIRs* in *P. leptostachya*

Gene name	The open reading frame (bp)	Protein					Subcellular localization
		**Length (aa)**	**MW (kDa)**	**pI**	**Signal peptide (aa)**	**N-Glyc (Asn-X-Thr/Ser) position**	
*PlDIR1*	561	187	20.97	7.04	1–22	52, 65, 122, 140	Chloro. Extracellular
*PlDIR2*	561	187	20.93	7.55	1–22	52, 65, 122, 140	Chloro. Plasma Membrane
*PlDIR3*	591	197	21.59	4.67	1–30	61, 189	Chloro. Plasma Membrane
*PlDIR4*	609	203	21.84	4.43	1–20	51, 122, 194	Chloro. Plasma Membrane
*PlDIR5*	567	189	20.32	4.55	1–20	51, 122	Extracellular. Plasma Membrane
*PlDIR6*	600	200	21.93	5.27	1–16	67, 173, 189	Chloro. Plasma Membrane
*PlDIR7*	570	190	21.09	5.3	1–20	36, 59	Chloro
*PlDIR8*	603	201	21.88	5.19	1–24	5, 193	Chloro. Extracellular
*PlDIR9*	606	202	22.16	8.07	-	59, 143	Cytoplasmic. Nuclear
*PlDIR10*	573	191	21.22	10.08	1–27	130	Chloro. Mitochondrial
*PlDIR11*	600	200	21.95	5.67	1–23	53, 167	Extracellular. Plasma Membrane
*PlDIR12*	603	201	21.51	9.26	1–20	-	Extracellular
*PlDIR13*	543	181	19.78	10.13	-	32, 77, 173	Chloro. Plasma Membrane
*PlDIR14*	555	185	20.06	8.46	1–19	124, 167	Chloro. Extracellular
*PlDIR15*	603	201	21.95	9.63	-	31, 69, 82, 103	Cytoplasmic. Plasma Membrane

By using the WoLF PSORT and CELLO subcellular localization software, PlDIRs were predicted to be mainly located in the chloroplast (chloro), plasma membrane, and extracellular. A total of 10 PlDIRs (PlDIR1-4, 6–8, 10, 13, and 14) were located in the chloro. Among these, five PlDIRs (PlDIR2, 3, 4, 6, and 13) were also located in the plasma membrane, three (PlDIR1, 8, and 14) were also located in extracellular space, and PlDIR10 was also located in mitochondria. Three PlDIRs (PlDIR5, 11, and 12) were distributed extracellularly, and two (PlDIR5 and 11) were also located in the plasma membrane. In addition, PlDIR9 and PlDIR15 were distributed in the cytoplasm, and also located in the nuclear and plasma membranes, respectively (Table 1).

### Phylogenetic analysis and classification of *PlDIRs*

**Fig. 1:**
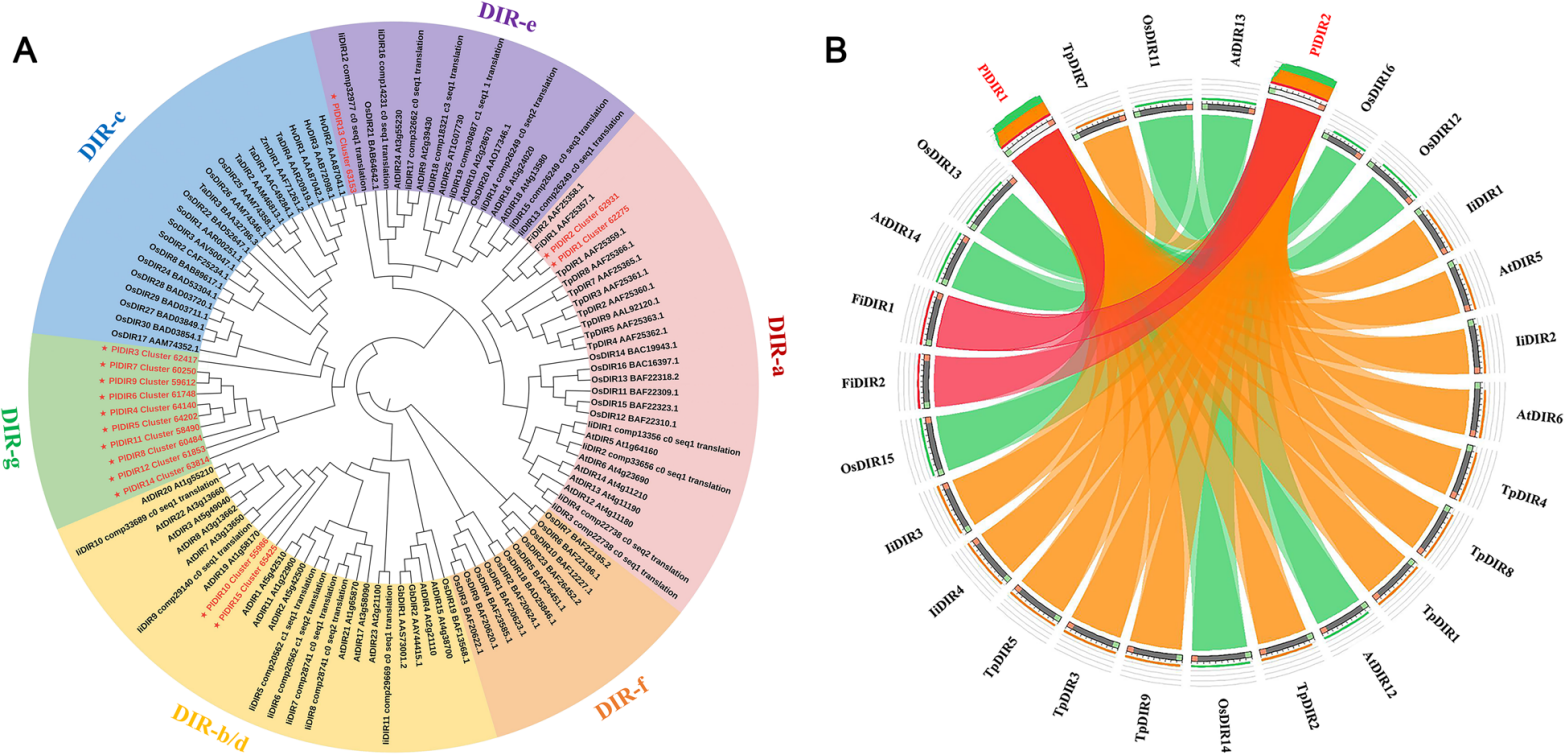
Phylogenetic relationships of DIRs from *P. leptostachya* and other plant species. **A**. Phylogenetic tree of 15 DIRs from *P. leptostachya* and other DIRs. Different groups of DIRs are indicated by different colors. PlDIRs are written in red and labeled with a red star. **B**. Circoletto radial diagram with ribbons connecting the PlDIRs and DIR orthologs in DIR-a subfamily. The colors of the ribbons are relative to the best BLAST alignment score, with matches within 80% of the best match as red, within 60% as orange, and within 40% as green. Light grey (PlDIRs) and dark grey bands on the periphery of the diagram represent the protein sequences, with the start and end of the sequence shown as green and red blocks, respectively. Ribbons representing the best hits are outlined and placed on top of all other ribbons

To further group and predict the potential functions of PlDIRs from well-studied DIRs in other plants, a phylogenetic tree was constructed with the amino acid sequences of PlDIR1-15 and 97 previously characterized DIRs from *A. thaliana*, *O. sativa*, *I. indigotica*, and other selected plant species. A total of 112 DIR/DIR-like proteins were categorized into six well-conserved subfamilies: DIR-a, b/d, c, e, f, and g (Fig. 1A). PlDIR members were grouped into four subfamilies (DIR-a, b/d, e, and g); DIR-c was a monocot specific subfamily, no proteins from our study were clustered into this DIR group. Ten members of PlDIRs (PlDIR3/4/5/6/7/8/9/11/12/14) were uniquely clustered into subfamily DIR-g. Two PlDIRs (PlDIR1/2) were clustered into DIR-a with two *F. intermedia*, eight *Thuja plicata*, six *O. sativa*, five *A. thaliana,* and four *I. indigotica* proteins. Another two PlDIRs (PlDIR10/15) were clustered into DIR-b/d with fourteen *A. thaliana*, seven *I. indigotica*, two *Gossypium barbadense*, and one *O. sativa* protein. PlDIR13 was clustered into DIR-e with eight *I. indigotica*, six *A. thaliana*, and two *O. sativa* proteins.

Previous studies have demonstrated that the DIR-a group members AtDIR5/6, TpDIR5/8, and FiDIR1 were involved in the formation of ( ±)-pinoresinol [20, 41, 42]. PlDIR1 and PlDIR2 belong to the DIR-a subfamily. To predict the potential functions of these two proteins, Circoletto was used to identify and visualize the sequence similarities between them and other members in DIR-a. As the results showed, PlDIR1 and PlDIR2 exhibited the highest sequence identity with FiDIR1 and FiDIR2, suggesting their roles in the lignan biosynthesis process (Fig. 1B). In addition, a comparison of PlDIR protein sequences shows that the protein similarity ranges from 18.6 to 93.6%, indicating the functional diversity among PlDIRs. The sequence similarity of PlDIR1 and PlDIR2 proteins in the DIR-a subfamily is exceptionally high, at 93.6%, whereas DIR-e member PlDIR13 exhibits low sequence similarity with other PlDIR proteins (Additional file 1: Table S1).

### Protein characterization and tertiary structures of PlDIRs

**Fig. 2:**
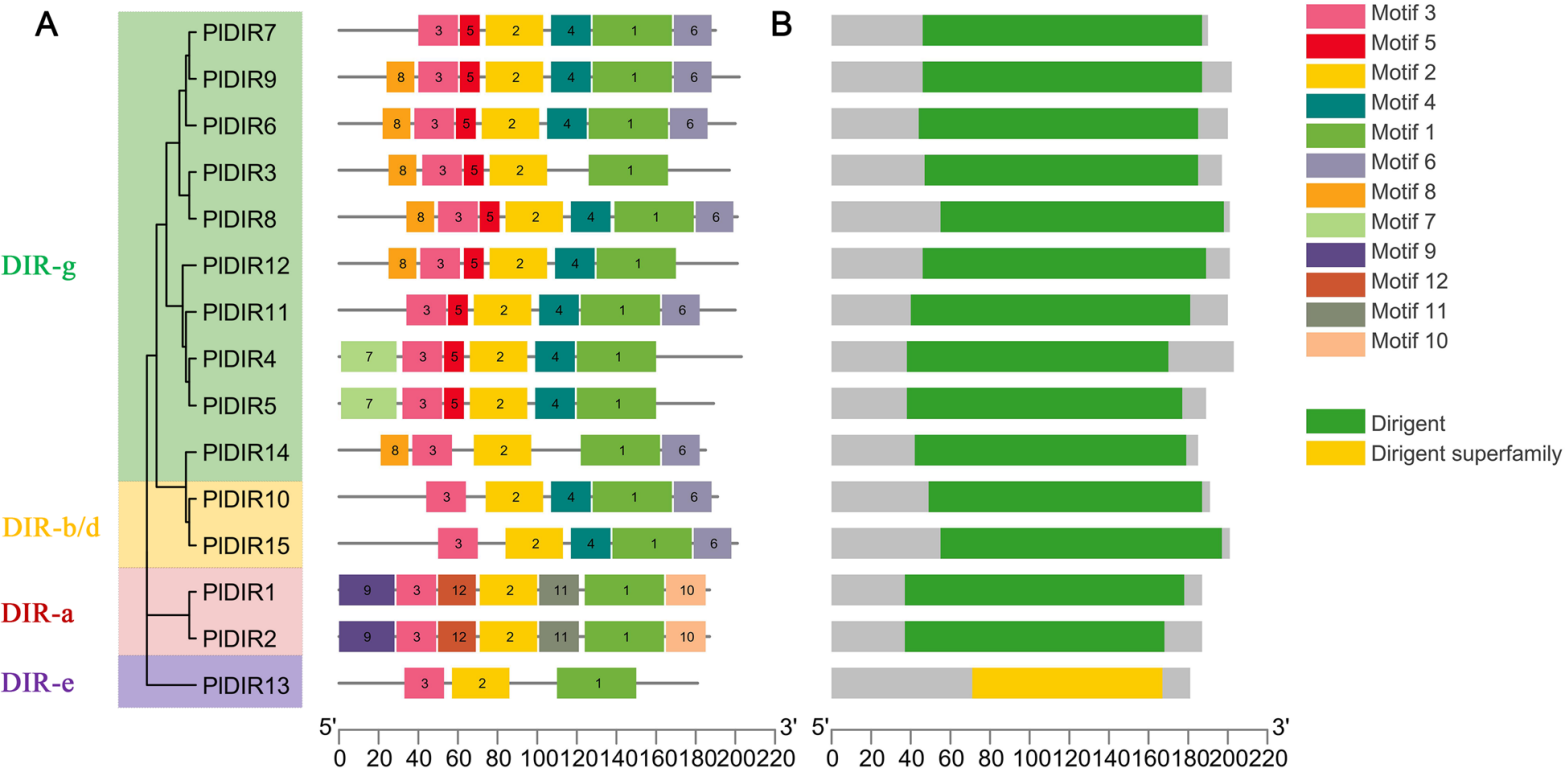
Distribution of conserved motifs and domains of PlDIRs. **A**. Distribution of conserved motifs in PlDIRs. Twelve putative motifs are shown in different colored boxes. The sequence information for each motif is provided in Additional file 2: Table S2.** B**. The position of the conserved DIR domain in each PlDIR protein

Twelve conserved motifs of PlDIR proteins were identified by MEME; the details were listed in Additional file 2: Table S2, and a schematic diagram was designed to characterize the structural diversity of the DIR proteins (Fig. 2A). There are 3–7 conserved motifs contained in all of the PlDIR proteins. The highly conserved motifs 1–3 were found in all subgroups and were present in fifteen sequences. Good distributions of motifs 4–6 were found in ten, nine, and eight proteins, respectively, excluding the members of DIR-b/d and g subfamilies. The majority of PlDIR members belonging to the same subfamily shared certain conserved motifs, illustrating the functional conservation within subfamilies as well as the variety within distinct subfamilies. For example, motifs 7, and 8 were only found in the DIR-g subfamilies, and motifs 9–12 were specifically present in the DIR-a subfamilies. In addition, the result of domain position analysis revealed that all of the conserved DIR domains were located close to the C terminal in the related proteins (Fig. 2B).

**Fig. 3:**
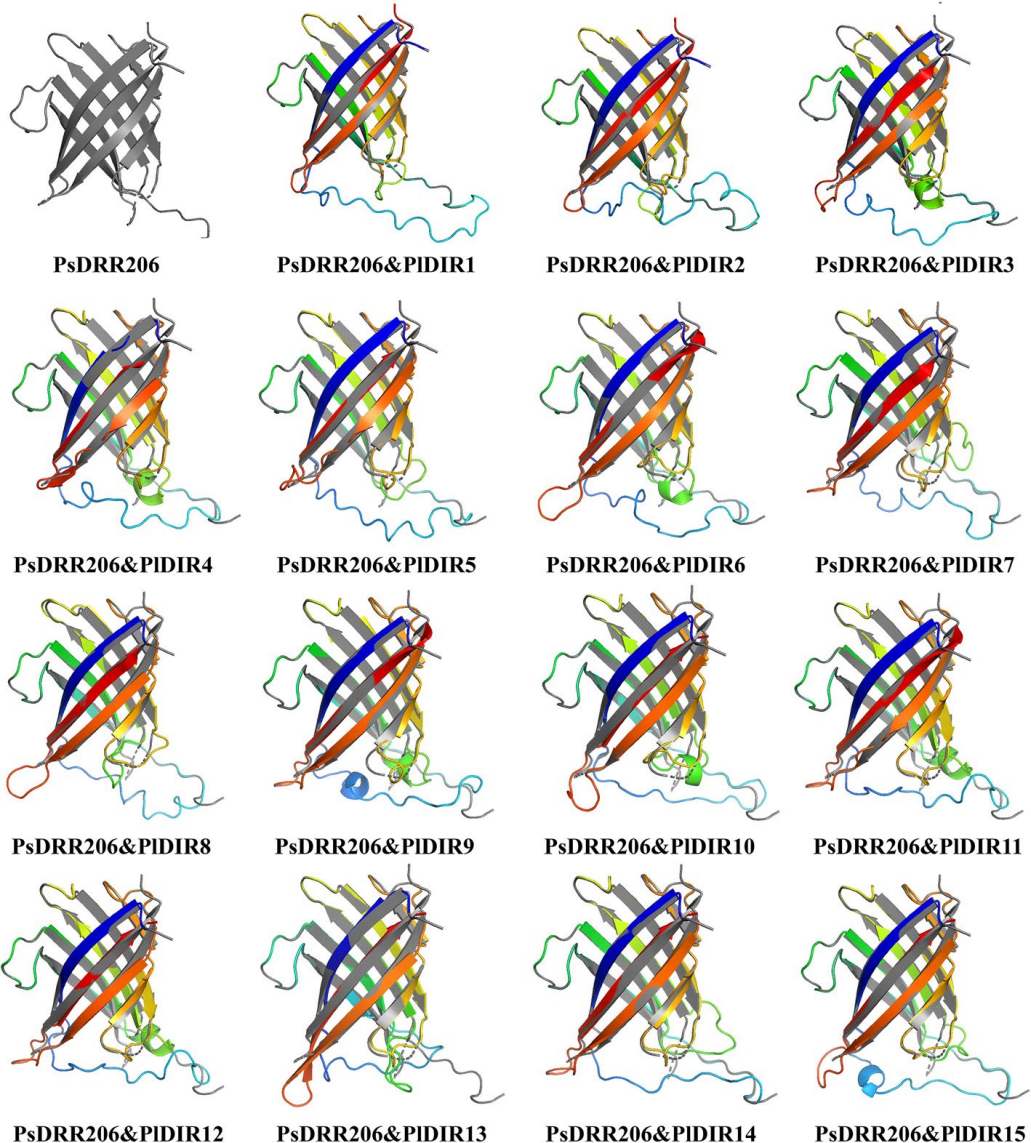
Predicted tertiary structures of PlDIR proteins. The prediction of PlDIRs was compared and merged with PsDRR206 (associated with ( +)-pinoresinol)

In addition, the monomer of the pea (*Pisum Sativum*) DIR protein PsDRR206 (C4REV.A) associated with ( +)-pinoresinol was used as the template [43], which shared 25–59% sequence identity with the PlDIRs, to build the 3D structures of 15 PlDIR proteins. As Fig. 3 shows, after comparing and merging PlDIRs with PsDRR206, the 3D structures of PlDIR1 and 2 could be well integrated with PsDRR206, indicating their similarity in structures or even in functions.

### Expression patterns of *PlDIR* genes in different tissues

**Fig. 4:**
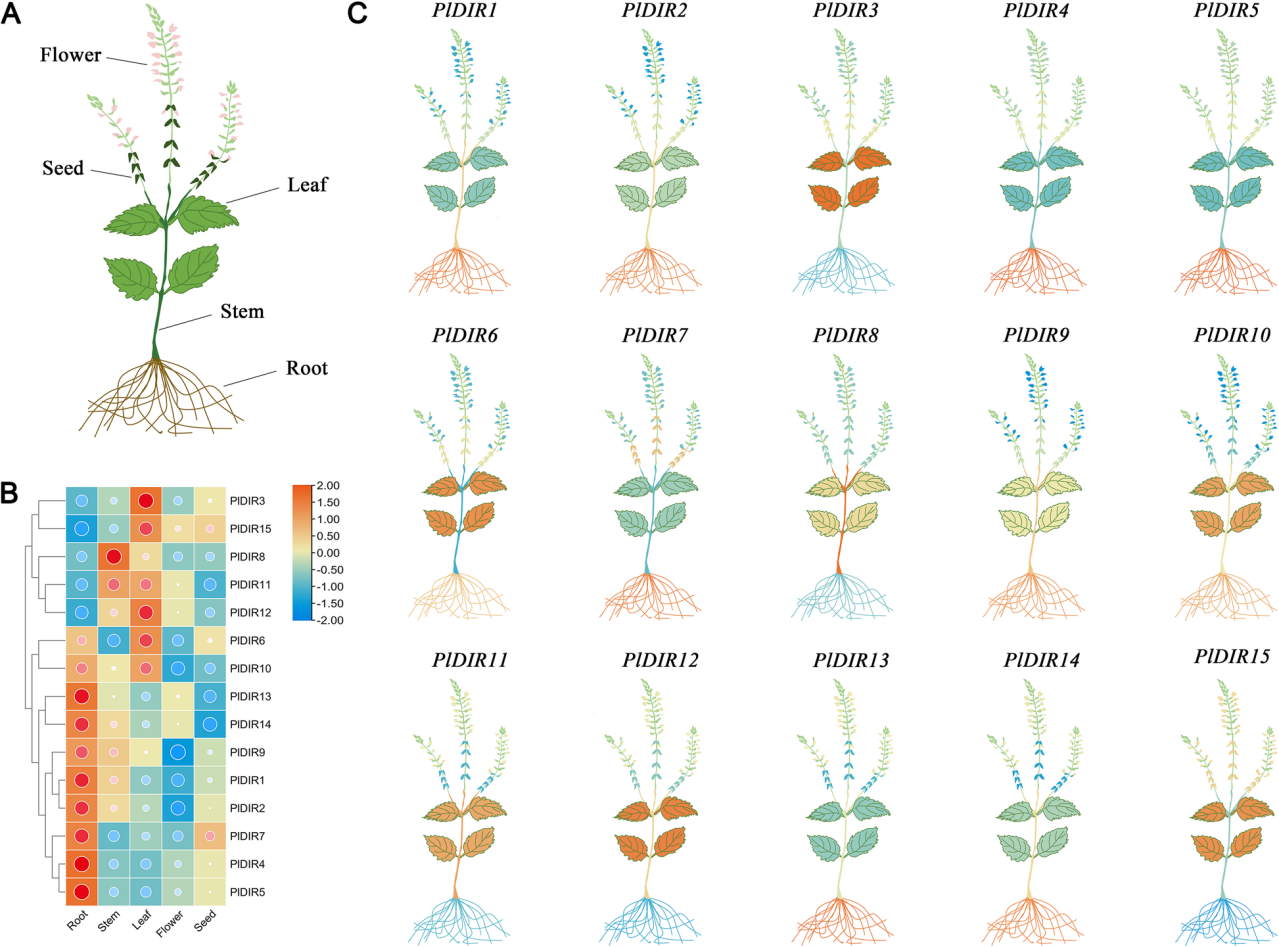
Expression patterns of *PlDIR* genes in various tissues (root, stem, leaf, flower, and seed). **A**. Diagram showing the different tissues of the *P. leptostachya* plant. **B**. The heatmap was drawn by TBtools using mean values.** C**. The expression patterns of *PlDIR* genes are presented by a cartoon heatmap. The data were normalized with the expression level of *Pl5.8 s RNA* in the root by the 2^−ΔΔCt^ method. Color orange represents a high expression level and blue represents a low expression level

Because the transcript abundance of a gene could reflect its function to a certain degree, the relative expression level of the 15 *PlDIRs* was analyzed in the tissues of the root, stem, leaf, flower, and seed by quantitative real-time reverse transcription-PCR (qRT-PCR). The results were presented in the form of *P. leptostachya* cartoon heatmaps (Fig. 4), and the expression trends were clustered in Fig. 4B. Based on the heatmap analysis, most *PlDIRs* have a comparatively higher transcript abundance in roots, leaves, and stems than in seeds and flowers. 8 *PlDIRs* (*PlDIR1*/*2*/*4*/*5*/*7*/*9*/*13*/*14*) displayed the highest transcript abundance in root tissues, and a higher level of expression was observed in leaf tissue for 5 *PlDIRs* (*PlDIR3*/*6*/*10*/*12*/*15*). *PlDIR8* and *11* showed specific higher transcript abundance in the stems, and *PlDIR7* was the only one that showed an accumulated expression level in seeds. However, all of these genes were hardly expressed in flowers.

### Expression responses of *PlDIRs* gene to signaling molecules

**Fig. 5:**
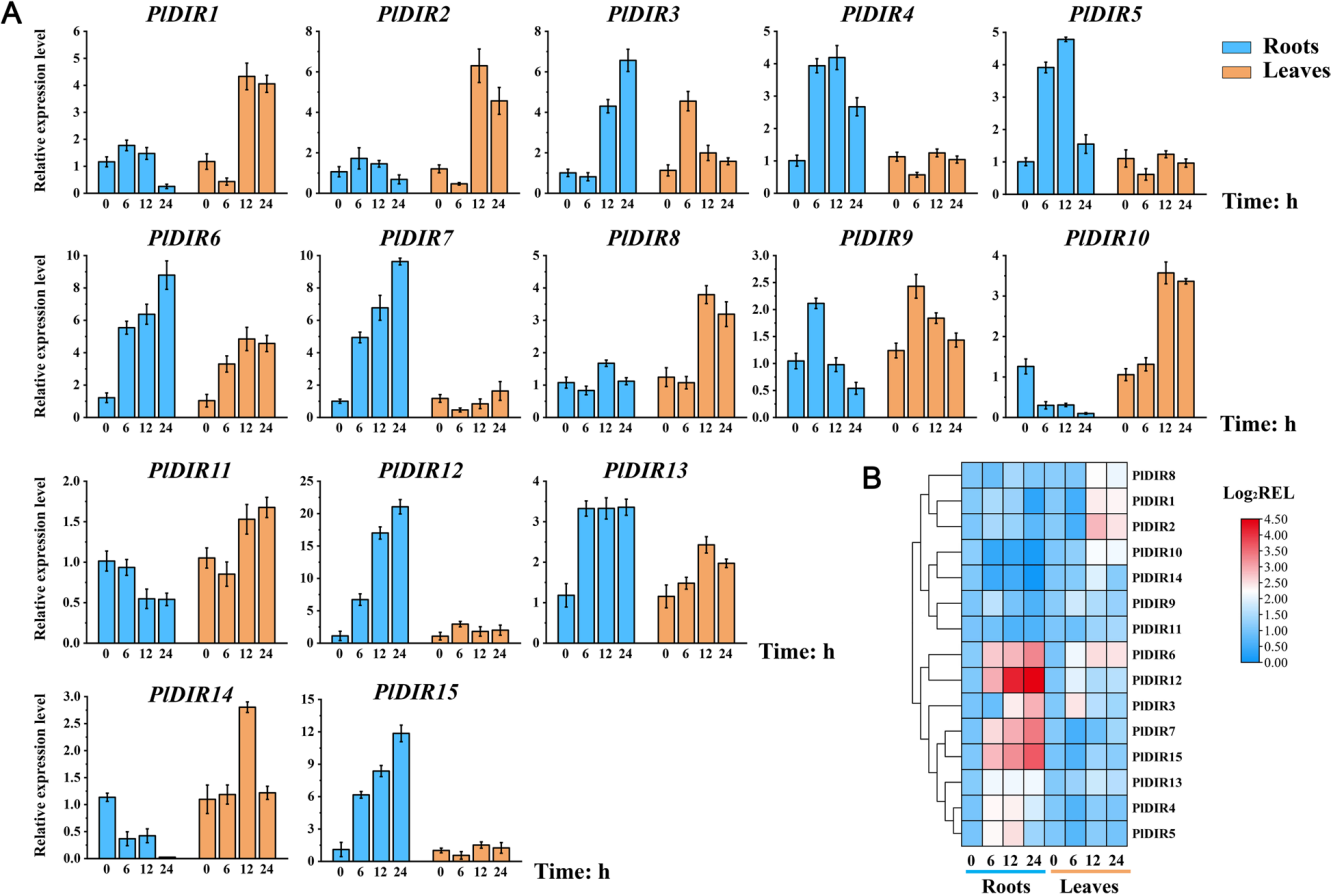
Relative expression level of *PlDIR* genes under MeJA treatment. The data were normalized with the expression level of 0 h by the 2^−ΔΔCt^ method. Error bars represent the mean ± standard deviation (SD) of 3 biological replicates. The color red in the heatmap represents a high expression level, and blue represents a low expression level

**Fig. 6:**
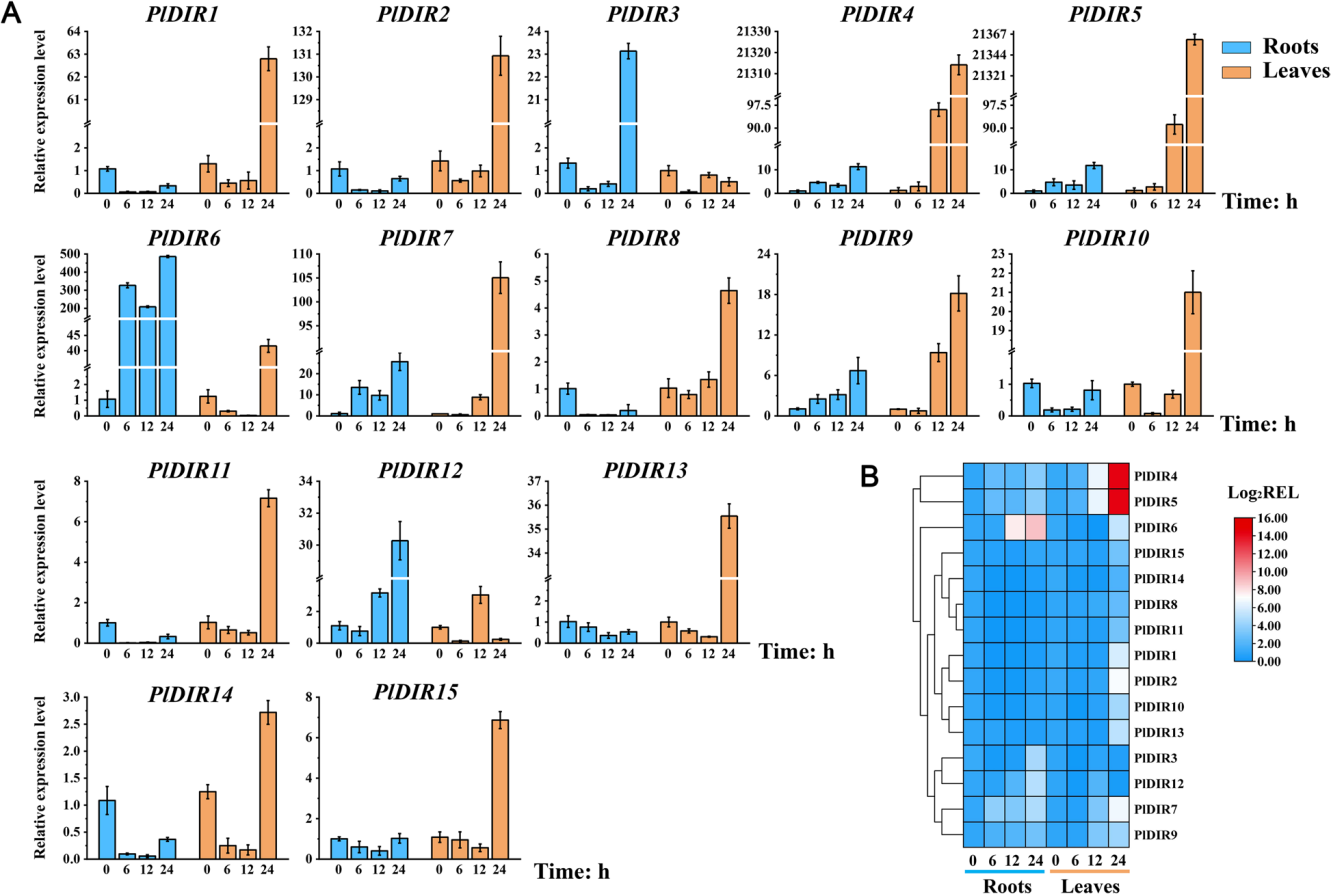
Relative expression level of *PlDIR* genes under SA treatment. The data were normalized with the expression level of 0 h by the 2^−ΔΔCt^ method. Error bars represent the mean ± standard deviation (SD) of 3 biological replicates. The color red in the heatmap represents a high expression level, and blue represents a low expression level

**Fig. 7:**
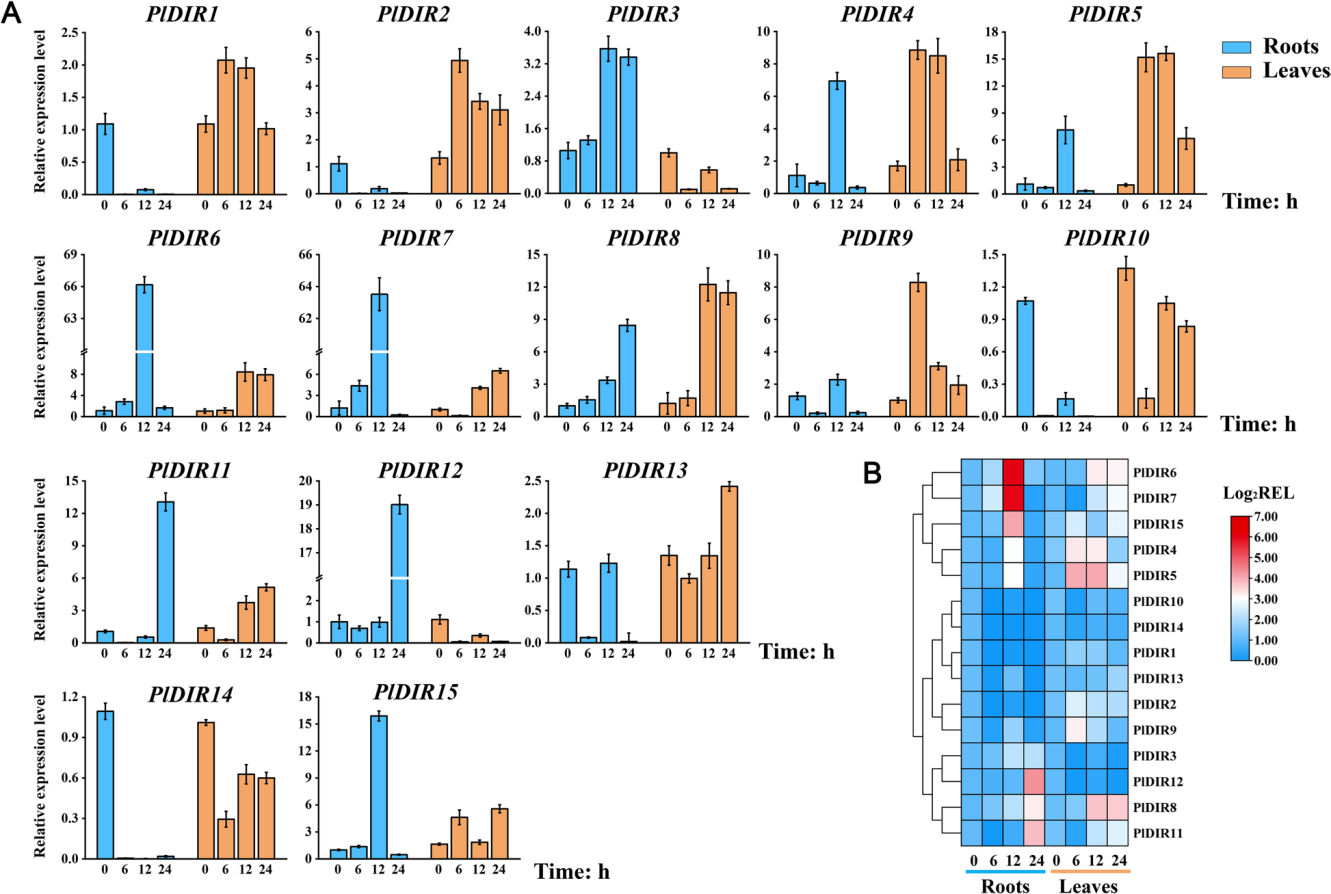
Relative expression level of *PlDIR* genes under ETH treatment. The data were normalized with the expression level of 0 h by the 2^−ΔΔCt^ method. Error bars represent the mean ± standard deviation (SD) of 3 biological replicates. The color red in the heatmap represents a high expression level, and blue represents a low expression level

Based on the tissue higher expressions of *PlDIRs*, roots, and leaves of *P. leptostachya* were selected to further analyze the response patterns of *PlDIRs* genes to three stress-related signaling molecules (MeJA, SA, and ETH) at 0, 6, 12, 24 h by qRT-PCR (Figs. 5, 6 and 7). The results showed that the majority of relative expression levels of *PlDIRs* were upregulated, but the response time and fold upregulation were inconsistent. For MeJA treatment, the response patterns of *PlDIR1*/*2*/*9* were similar. Their relative expression in leaves was higher than in roots and reached a maximum of 6 h in roots. *PlDIR10*, *PlDIR11,* and *PlDIR14* have similar expression profiles, which are suppressed in roots and more sensitive to MeJA in leaves. In addition, eight of the *PlDIRs* showed significant responses in roots, when compared to those in leaves. Among them, *PlDIR4* and *PlDIR5* showed higher expression levels at 12 h in roots; *PlDIR3*/*6*/*7*/*12*/*15* reached a maximum at 24 h, and the expression level was up-regulated by more than 6, 8, 9, 20, and 10 folds, respectively (Fig. 5).

After exposure to SA treatment, 12 of the 15 *PlDIRs* were highly expressed to the maximum at 24 h in leaves, except for *PlDIR3*, *PlDIR6,* and *PlDIR12*, which reached their expression peaks at 24 h in roots. PlDIR4/5 has a strong response to SA in leaves compared with other genes, with the upregulation occurring more than 20,000 times. Moreover, the expression of *PlDIR1*/*2*/*8*/*10*/*11*/*13*/*14*/*15* was suppressed in roots at all the tested time points; and *PlDIR4*/*5*/*7*/*9* has similar expression profiles, which increased by more than sixfold in roots at 24 h (Fig. 6).

The ETH treatment induced the expression of PlDIR6/7/15 in roots much faster than in leaves, with peaks at 12 h. Especially for *PlDIR6*/*7*, which increased by more than 60 folds. *PlDIR11* and *PlDIR12* were then upregulated more than 12 times in 24 h. On the contrary, *PlDIR1*, *PlDIR2*, *PlDIR10*, and *PlDIR14* were down-regulated at different time points in the roots. After the treatment with ETH, the expression profile of *PlDIR1*/*2*/*4*/*5*/*8*/*9*/*13* in leaves is much higher than that in roots, which reached a maximum at 6 h or 12 h, except for *PlDIR13* (Fig. 7)*.*

### Amino acid sequence alignments of DIR-a subfamily

**Fig. 8:**
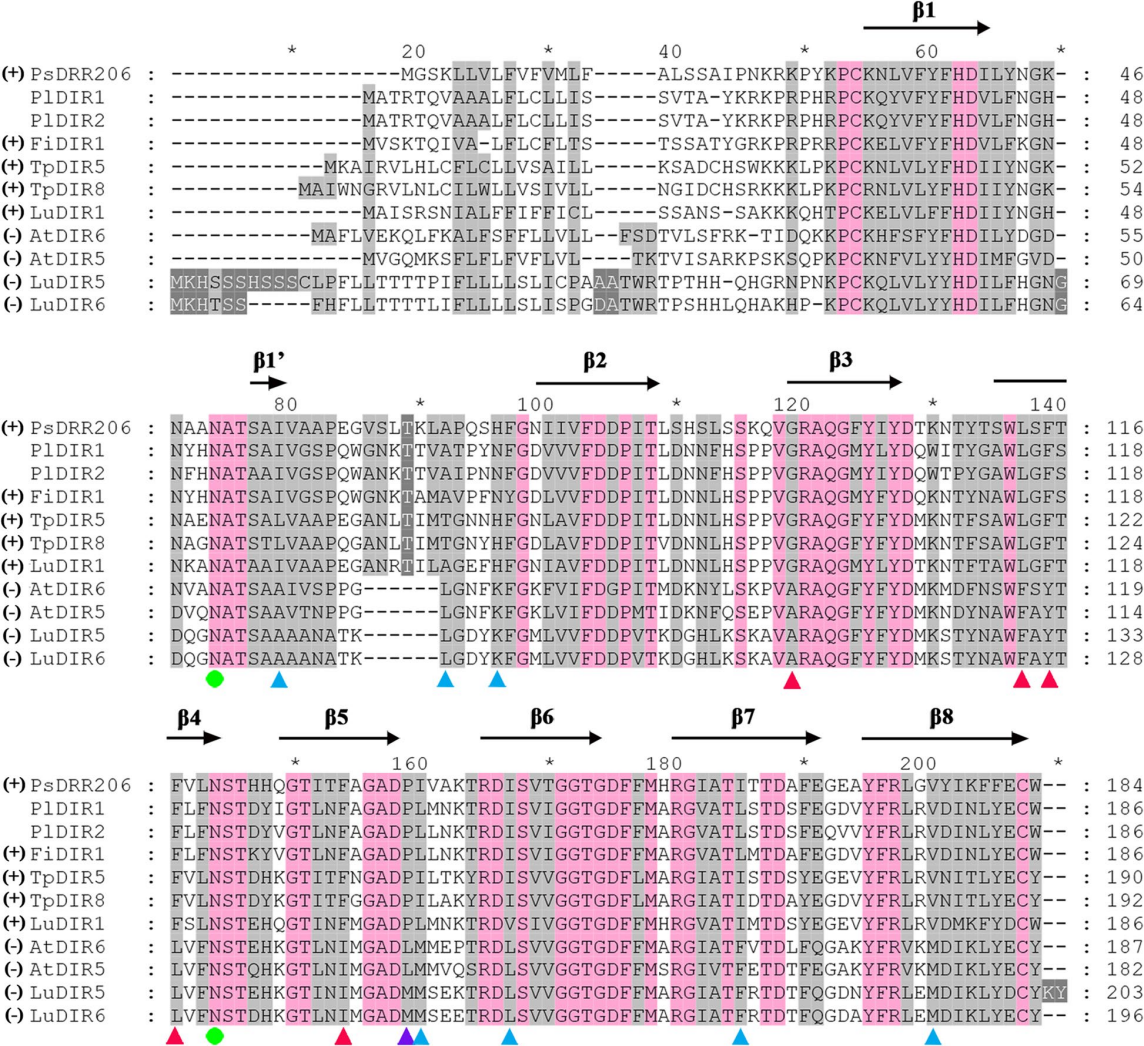
The alignment of PlDIR1 and PlDIR2 protein sequences with other ( +)- and (-)-DIRs. Only full-length sequences are shown. Strictly conserved residues are boxed in pink. Those that are conserved in ( +)- and (-)-DIRs are highlighted by purple and blue triangles, respectively. Red triangles indicate residues that are differentially conserved in ( +)- and (-)-DIRs. *N*-glycosylation sites are shown by green circles. Secondary structure elements are shown above the alignment

Phylogenetic analysis revealed that two PlDIRs (PlDIR1 and PlDIR2) were DIR-a subfamily members, and amino acid sequence alignments of them were performed to determine if any hypothetical functions could be inferred (Fig. 8). According to a previous study, the DIR-a subfamily members AtDIR5 and AtDIR6 from *A. thaliana*, LuDIR5, and LuDIR6 from *L. usitatissimum* were found to guide *E*-coniferyl alcohol to form (-)-pinoresinol [41, 44]. However, in the presence of PsDRR206 from *P. sativum*, FiDIR1 from *F. intermedia*, TpDIR5 and TpDIR8 from *T. plicata,* and LuDIR1, the final product of *E*-coniferyl alcohol was the enantiomer ( +)-pinoresinol [20, 42–44]. As shown in Fig. 8, an eight-stranded antiparallel β-barrel (black arrow) and two *N*-glycosylation sites at aa 74 and 144 (Asn; green circles) were presented in all protein sequences. Strictly conserved residues (pink box) are conserved in all characterized pinoresinol-forming DIRs [45, 46]. Five differentially conserved residues at aa 119, 137, 139, 141, and 154 were involved in forming ( +)-pinoresinol or (-)-pinoresinol (red triangle). Furthermore, conserved residues at aa 79, 92, 96, 160, 167, 185, and 200 (blue triangle) are involved in forming (-)-pinoresinol, whereas DIR residues at aa 159 (purple triangle) are for forming ( +)-pinoresinol. The key amino acid residues of PlDIR1 and 2 are highly consistent with the ( +)-pinoresinol forming DIR proteins, indicating their functions in catalyzing the generation of ( +)-pinoresinol rather than (-)-pinoresinol.

### Heterologous expression and catalytic activities of recombinant PlDIR1 protein

**Fig. 9:**
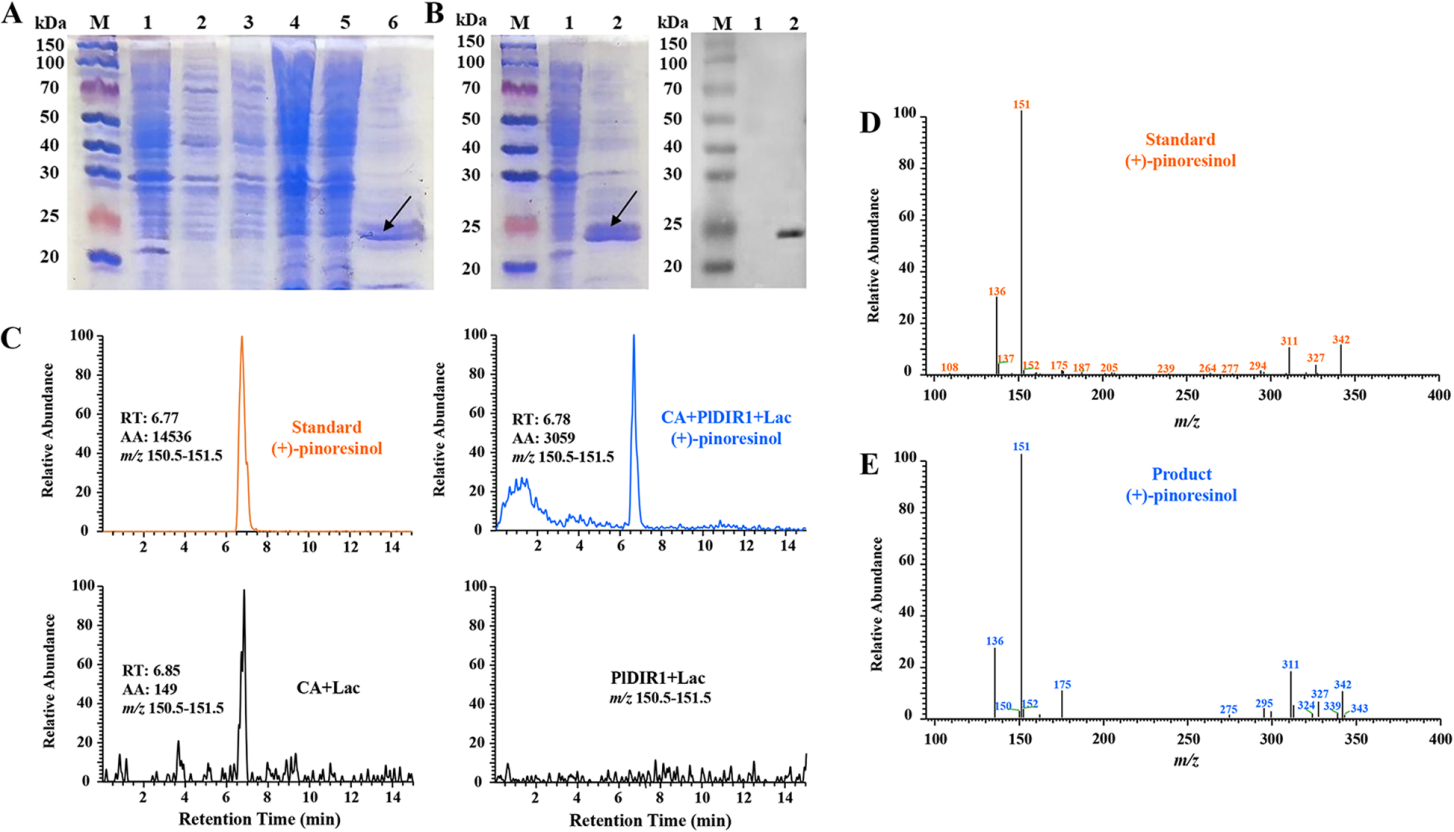
Analysis of PlDIR1 dirigent activity by LC–MS/MS. **A**. Expression of recombinant PlDIR1 protein in *E. coli* BL21 (DE3) was induced using 0.1 mM IPTG at 16℃ for 6–15 h and purified from the soluble fraction of the induced cells using resin with an affinity for the His-Tag. M: protein marker; Lane 1: the empty vector pET-29a( +) expressed in *E. coli* BL21 (DE3) and induced by IPTG for 6 h; Lane 2–5: pET29a( +)-PlDIR1 induced by IPTG for 6, 9, 12, and 15 h; Lane 6: purified soluble PlDIR1 protein. **B**. Western Blot assay of the recombinant PlDIR1 protein. M: protein marker; Lane 1: the empty vector pET-29a( +) expressed in *E. coli* BL21 (DE3) and induced by IPTG for 6 h; Lane 2: purified soluble PlDIR1 protein. **C**. LC–MS/MS analysis of ( +)-pinoresinol in the catalytic product of the recombinant PlDIR1 protein. Extracted ion chromatograms show the intense peak of standard ( +)-pinoresinol or catalytic product of PlDIR1 at *m/z* = 150.5–151.5. D, E. Mass spectra of standard ( +)-pinoresinol and catalytic product of PlDIR1. RT, retention time; AA, area

After induction for 6–15 h at 16 °C with 0.1 mM IPTG, the recombinant PlDIR1 protein with a MW of around 20.97 kDa were maximally expressed in *E. coli* BL21 (DE3) at 12 and 15 h. The recombinant protein with an *N*-His_6_-tag was purified by a Ni–NTA affinity column and verified by SDS-PAGE and Western blotting (Fig. 9A, B and Additional file 3: Fig. S1). Western blot detection showed that the PlDIR1 could specifically combine with anti-His-tag antibodies. One single immunoreactive band was detected from the recombinant PlDIR1 protein, and no such band was found in the empty vector pET-29a( +) (Fig. 9B). Then, in vitro enzyme activity assays were conducted to determine the potential catalytic activity of the recombinant protein, and reaction products were analyzed by LC–MS/MS. As seen in Fig. 9C, when substrate and laccase (Lac) protein were provided to recombinant protein PlDIR1, a peak at 6.78 min was observed (*m/z* 150.5–151.5), which was identical to the peak observed in chromatograms generated from standard ( +)-pinoresinol (*m/z* 150.5–151.5). However, in reaction samples without PlDIR1 (contains *E*-coniferyl alcohol and Lac) or substrate *E*-coniferyl alcohol (contains PlDIR1 and Lac), no such peak was detected. Moreover, the ion fragments observed in the mass spectrum for the peak appearing at 6.78 min in the PlDIR1-containing assays (Fig. 9E) were consistent with the fragmentation of the ( +)-pinoresinol standard (Fig. 9D). These results illustrate that PlDIR1 could catalyze the conversion of *E*-coniferyl alcohol to ( +)-pinoresinol with the help of Lac.

### The correlation between hormone-induced lignan accumulations and *PlDIR1* expression profile in *P. leptostachya* roots

**Fig. 10:**
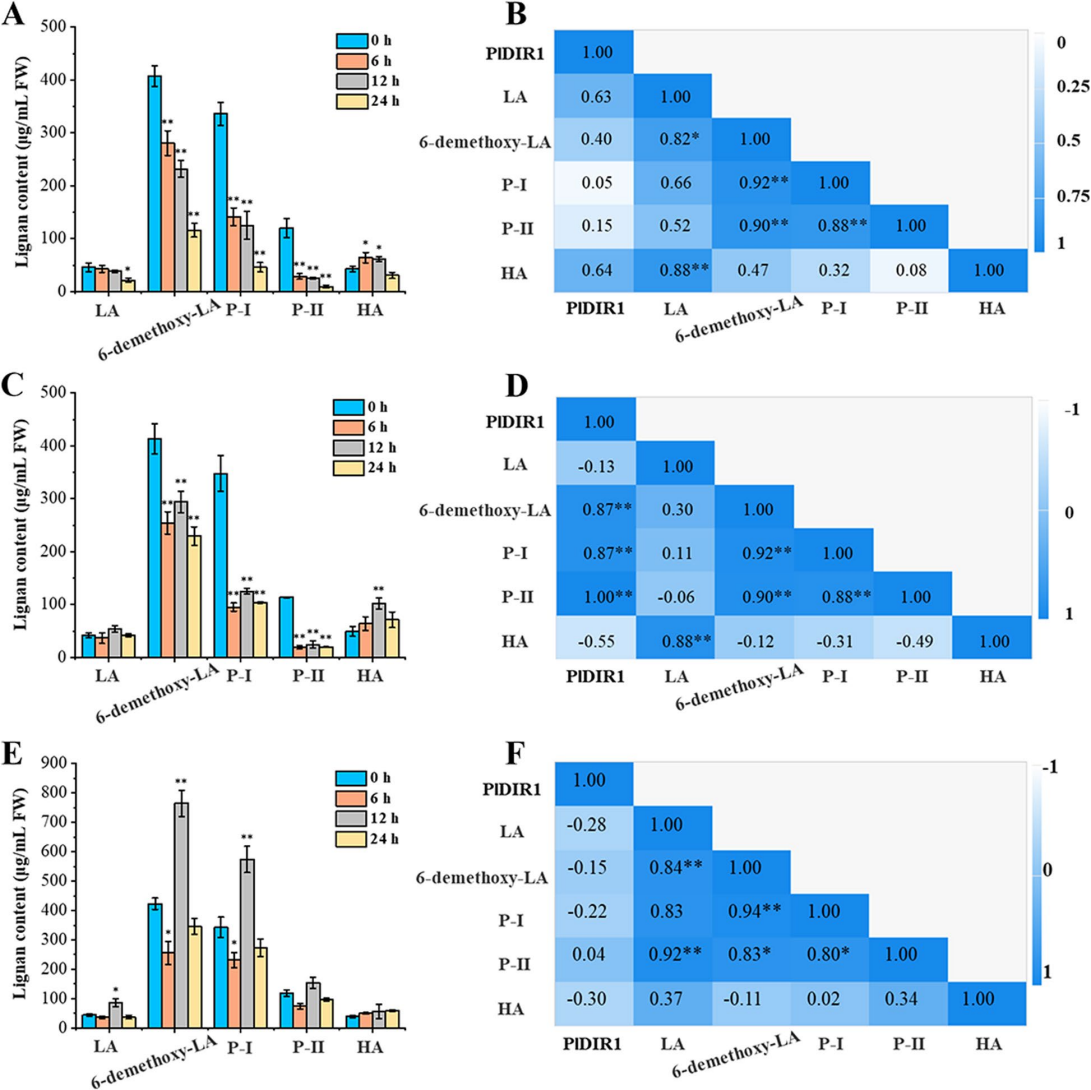
The correlation analysis between hormone-induced lignan accumulations and *PlDIR1* expression profile. **A**, **C**, **E**. Effect of MeJA, SA, ETH treatments on the accumulation of five key lignans in *P. leptostachya* roots, respectively. **B**, **D**, **F**. The Pearson’s correlation coefficient between lignan contents and *PlDIR1* expression profile under MeJA, SA, ETH treatment, respectively. The number -1 to 1 indicates the correlation from low to high. Asterisks indicate the significant correlation (*P < 0.05, **P < 0.01, Student’s t-test); FW, fresh weight

The catalytic process of *PlDIR1* exists upstream of the *P. leptostachya* lignan biosynthesis pathway. To determine the relationship between *PlDIR1* and other metabolites in this pathway, a correlation analysis using Pearson’s correlation coefficient (PCC) was performed to identify possible correlations between the *PlDIR1* expression and the investigated metabolites under hormone treatments at different time points. This data was visualized as a heat map. To achieve this purpose, the accumulation of five key lignan compounds (Leptostachyol acetate, LA; 6-Demethoxy-leptostachyol acetate, 6- demethoxy-LA; P-I, P-II, and HA) in *P. leptostachya* roots was firstly analyzed by HPLC, since they are mainly stored in root tissue. As a result, after MeJA treatment, only HA showed a sightly accumulation at 6 and 12 h; the contents of the remaining metabolites were reduced significantly compared to the control and reached the lowest levels at 24 h (Fig. 10A). Considering the correlation coefficients between *PlDIR1* transcript levels and accumulation of five metabolites were 0.63, 0.40, 0.05, 0.15, and 0.64, respectively, *PlDIR1* is correlated with LA and HA, but not or minimally correlated with 6-demethoxy-LA, P-I, and P-II (Fig. 10B). A similar trend was found for lignan accumulation under SA treatment (Fig. 10C), but dramatic correlations (*P* < 0.01) were presented between *PlDIR1* expression profiles and the contents of 6-demethoxy-LA, P-I, and P-II, with correlation coefficients of 0.87, 0.87, and 1, respectively (Fig, 10D). Different results were presented after ETH treatment: four metabolites (LA, 6-demethoxy-LA, P-I and II) showed the highest abundance at 12 h with varying degrees, while HA content was not influenced (Fig. 10E). Moreover, the expression of *PlDIR1* was not related to metabolites induced by ETH, as revealed by the PCC analysis in Fig. 10F.

### Docking analysis of substrate interactions

**Fig. 11:**
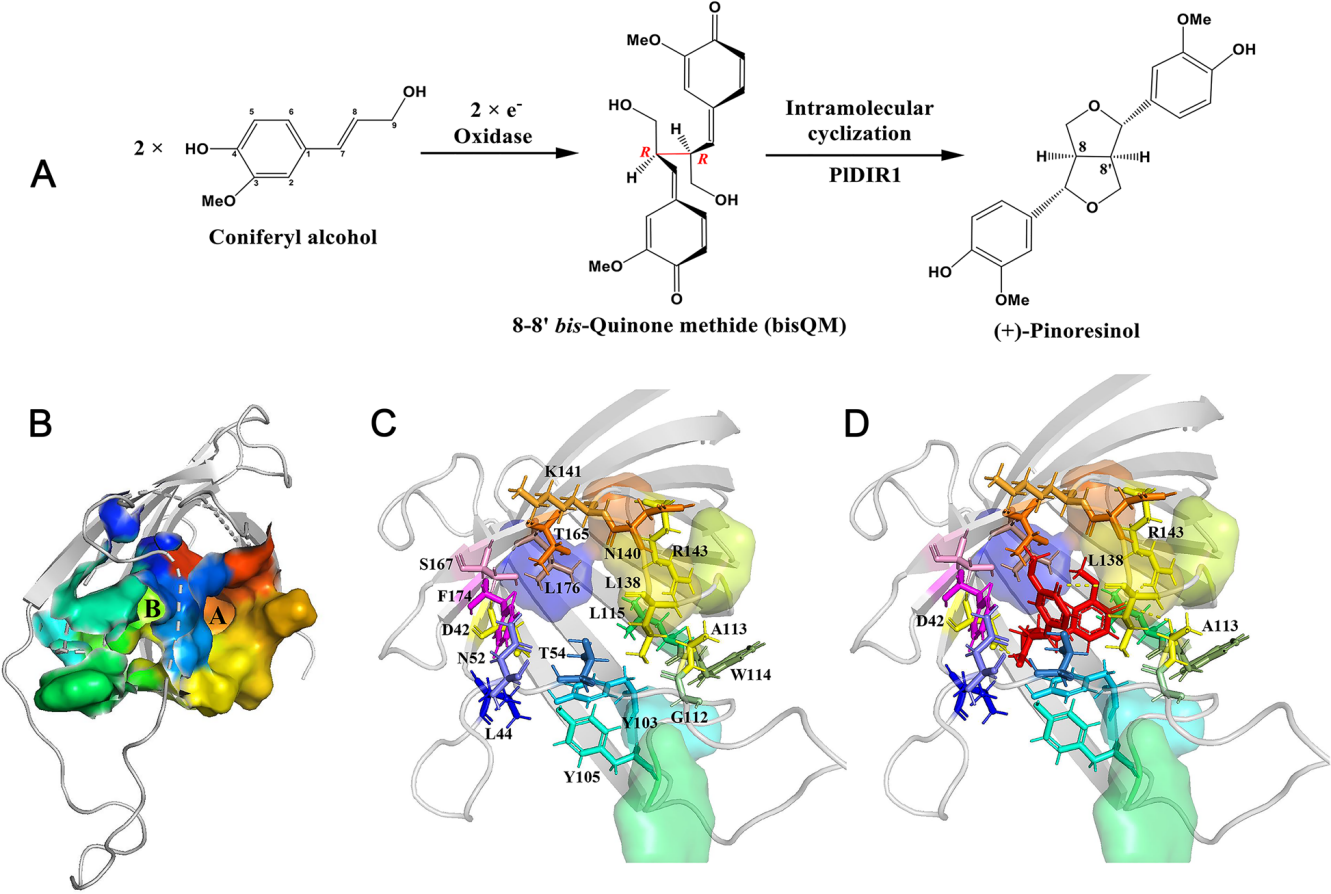
Molecular docking model for PlDIR1 protein with the proposed reaction intermediate for ( +)-pinoresinol. **A**. Putative biosynthesis mechanism to afford ( +)-pinoresinol. **B**. Surface representation of PlDIR1 showing the pockets A and B of the active sites. **C**. View of the active site showing important residues within the binding pockets. **D**. Potential binding mode of bisQM (red molecular) in the active site of PlDIR1. Conformation and position were optimized by energy minimization after manual placement of the ligand. Possible hydrogen bonds are indicated with yellow dotted lines

To examine the enzymatic structure–function relationships underlying the ( +)-pinoresinol formation activity of PlDIR1, a molecular docking analysis was performed to gain some insight into the potential reaction mechanism involved. The homology model for PlDIR1 was generated based on the crystal structure reported for PsDRR206 [43]. As a result, two pockets (A and B) with different sizes were exhibited at the open end of the barrel of PlDIR1 (Fig. 11B), which is consumed to bind two substrate molecules. According to the docking studies for AtDIR6 and PsDRR206, the putative substrate for PlDIR1 is a reactive radical species, so it is hard to get the protein-substrate complexes straightforwardly. Accordingly, the bisQM, being the putative intermediate in pinoresinol formation following (CA·) radical coupling before cyclization of the furan rings, was used as a ligand to conduct the docking analysis (Fig. 11A). After docking runs, the one with the lowest energy and the greatest number of bindings was selected as the final analysis result. Important amino acids are present in the active site of PlDIR1 as previously reported in the structure and shown in Fig. 11C. Asp-42, Leu-44, Asn-52, Thr-54, Tyr-103, Tyr-105, Gly-112, Ala-113, Trp-114, Leu-115, Leu-138, Asn-140, Lys-141, Arg-143, Thr-165, Ser-167, Phe-174, and Leu-176 were the PlDIR1 amino acid residues that interacted with bisQM, and four hydrogen bonds were formed between Asp-42, Ala-113, Leu-138, Arg-143, and PlDIR1 (Fig. 11D).

### *PlDIRs* co-expression analysis with genes involved in lignan biosynthesis

**Fig. 12:**
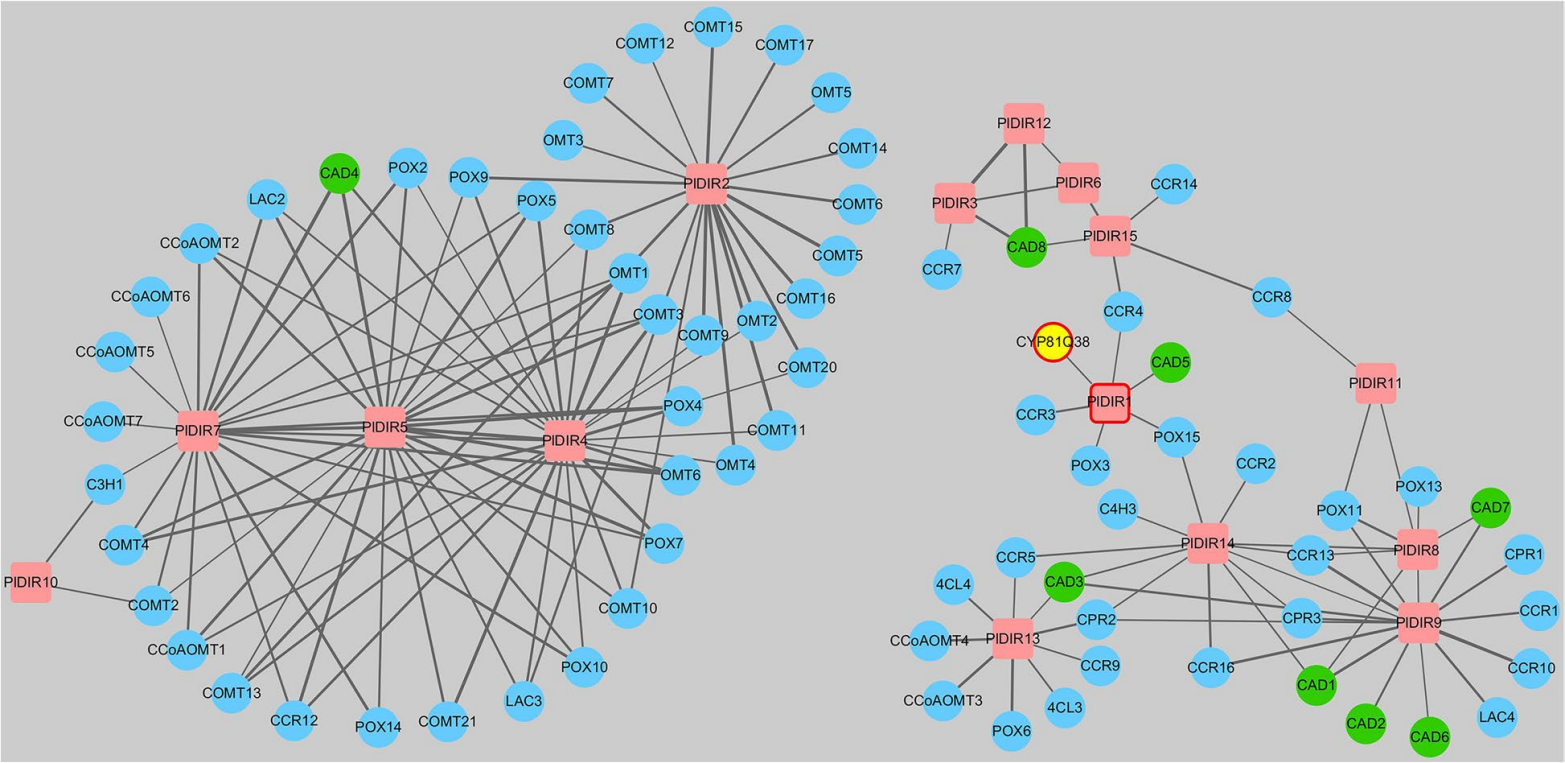
Co-expression correlations of genes involved in lignan biosynthesis. Edges are drawn when the linear correlation coefficient is > 0.7. Red rectangles represent PlDIRs; green circles represent characterized CADs; CYP81Q38 and PlDIR1 that might be involved in sesamin biosynthesis are marked with red circles. PAL, phenylalanine ammonia-lyase; C4H, trans-cinnamate 4-monooxygenase; C3H, *p*-coumarate 3-hydroxylase; COMT, caffeic acid 3-O-methyltransferase; 4CL, 4-coumarate-CoA ligase; CCoAOMT, caffeoyl-CoA O-methyltransferase; CCR, cinnamoyl-CoA reductase; CAD, cinnamyl-alcohol dehydrogenase; DIR, dirigent protein

To deepen the understanding of the *P. leptostachya* lignan biosynthesis pathway, *PlDIRs* and 108 lignan synthesis-related genes chosen from the *P. leptostachya* transcriptome were subjected to a co-expression analysis, which was generated with Cytoscape software. The selected genes are listed in Additional file 4: Table S3, and a schematic biosynthetic pathway is proposed in Additional file 5: Fig. S2 to gain insight into their position and potential roles. As shown in Fig. 12, a total of 87 co-expressed genes showed a greater correlation coefficient than 0.7 with at least one other gene.

A strong correlation was found between *PlDIRs* and lignan synthesis genes. Both peroxidase and laccase genes (*POXs* and *LACs*) are potentially involved in monolignol oxidation, i.e.: *PlDIR1* with *POX3* and *POX15*, *PlDIR2* with *POX9* and *LAC3*, *PlDIR4* with *POX2*/*4*/*5*/*7*/*9*/*10* and *LAC2*/*3*, *PlDIR5* with *POX2*/*4*/*5*/*7*/*9*/*10*/*14* and *LAC2*/*3*, *PlDIR7* with *POX2*/*4*/*5*/*7*/*10*/*14* and *LAC2*, *PlDIR8* with *POX11*/*13*, *PlDIR9* with *POX11* and *LAC4*, *PlDIR11* with *POX11*, *PlDIR13* with *POX6*, and *PlDIR14* with *POX15*. Furthermore, according to the pathway of lignan biosynthesis, genes of trans-cinnamate 4-monooxygenase (C4Hs), *p*-coumarate 3-hydroxylase (C3Hs), caffeic acid 3-O-methyltransferase (COMTs), 4-coumarate-CoA ligase (4CLs), caffeoyl-CoA O-methyltransferase (CCoAOMTs), cinnamoyl-CoA reductase (CCRs), and cinnamyl alcohol dehydrogenase (CADs) were in the upstream of *PlDIRs*, a reported CYP450 gene *PlCYP81Q38* catalyzing ( +)-sesamin biosynthesis from ( +)-pinoresinol was in its downstream [18]. As the results indicated, *PlDIRs* were co-expressed with genes that were involved in the lignan biosynthesis pathway, which were the catalyzed genes of continuous two or three reactions. For example, *PlDIR1* was predicted to co-express with *CCR3*/*4*, *CAD5,* and *PlCYP81Q38*, *PlDIR3*/*8*/*9*/*14*/*15* with *CCRs* and *CADs*, *PlDIR4*/*5*/*7* with *CCoAOMTs*, *CCR12* and *CAD4*, *PlDIR10* with *C3H1* and *COMT2*, and *PlDIR13* with *4CLs*, *CCoAOMTs*, *CCRs* and *CADs* (Fig. 12).

## Discussion

Our understanding of plant growth and development in many plant species has considerably advanced as a result of the characterization of *DIR* genes over the past few decades. Plant DIR proteins are involved in both abiotic and biotic stress responses. They were first discovered for regio- and stereo-selective coupling in the process of lignan biosynthesis. Large multigene families made up of *DIRs* have been found in terrestrial plants, and different plant species have varying numbers and types of DIRs. For example, 35 DIRs have been identified in spruce (Picea spp.) [21], 25 DIRs in *Arabidopsis* [21], 19 DIRs in *I. indigotica* [27], 27 DIRs in *B. rapa* [28], 49 DIRs in *O. sativa* [26], 35 DIRs in Chinese White Pear (*Pyrus bretschneideri*) [47], 44 DIRs in flax (*L. usitatissimum*) [25], 24 DIRs in pepper (*Capsicum annuum* L.) [48], 45 DIRs in *M. truncatula* [24], 40 DIRs in *Populus trichocarpa* [49], 54 DIRs in soybean (*Glycine max*) [50] and 112 DIRs in Cucurbitaceae [51]. *P. leptostachya* is an important insecticidal plant and possesses essential insecticidal active ingredients (lignans), the characterization of *P. leptostachya DIR* genes would benefit the analysis of the formation process of lignan biosynthesis and stress defense in *P. leptostachya*. We used transcriptome-wide identification to characterize 15 *PlDIR* members and examined a variety of their traits in this study.

*N*-glycosylation is crucial for secretory proteins; it is a highly conserved and key Endoplasmic Reticulum post-translational modification with multiple functions during and after protein folding [52–56]. Approximately 80% of secretory pathway proteins are *N*-glycosylated at specific Asn residues [57]. In the current study, signal peptide sequences were presented in 80% of PlDIR proteins, which indicated that the majority of them traverse the secretory pathway. At the same time, the PlDIRs with peptide sequences also contain at least one *N*-glycosylation site (Table 1). In addition, the freshly produced protein can be directed to distinct subcellular compartments by the signal peptide [58], and our results from predicted subcellular localization revealed that PlDIR proteins were present in a variety of organelles, indicating they may be targeted for extracellular release or potential final localization in various cellular organelles (Table 1). Furthermore, PsDRR206 (C4REV.A), a ( +)-pinoresinol-formation protein with a characterized crystal structure, was utilized as the template to predict the homologous structures of PlDIRs. As a consequence, the barrel-like structure existed in all of the PlDIRs, among which PlDIR1 and 2 showed higher similarity in structures with PsDRR206 than others (Fig. 3).

DIRs from different species could be categorized into six well-known subfamilies (DIR-a, b/d, c, e, f, and g), according to phylogenetic analysis, although not all of the subfamilies are present in every species [21]. In this study, 15 PlDIRs were separated into four subfamilies, there were 2, 2, 1, and 10 members in DIR-a, DIR-b/d, DIR-e, and DIR-g, respectively (Fig. 1A). All investigations that have been published assume that DIRs in the DIR-a subclade specifically regulate the oxidative coupling of coniferyl alcohol in the production of ( +)- or (-)-pinoresinol. The DIR-a subfamily numbers (PlDIR1 and PlDIR2) of *P. leptostachya* are predicted to have the same function as other DIRs in this subfamily. According to current investigations, the stereoselective coupling reaction on the hemigossypol (substrate) to generate ( +)-gossypol in cotton species was characterized in a few members of the DIR-b/d subfamilies, such as GbDIR1 and 2 in *G. barbadense* [59], GhDIR4 in *Gossypium hirsutum*, and GaDIR1 in *Gossypium arboretum* [60]. Here, two PlDIRs (PlDIR10 and PlDIR15) were clustered into the DIR-b/d subfamily. The DIR-c is a subfamily that is only found in angiosperm monocots with low sequence conservation. Many members of the DIR-c subfamily have a distinctive amino acid extension at their C-terminus that is highly comparable to the jacalin-like domain found in lectin proteins. This extension may help members defend against biotic and abiotic stressors [21, 61]. None of the identified genes from *P. leptostachya* were clustered in this category. Additionally, it was believed that the DIR-e and DIR-f subfamilies were involved in lignin deposition in the Casparian strip [62] and defense against insects and wounding [21], respectively. Here, only one PlDIR (PlDIR13) was clustered in DIR-e and none in DIR-f. The DIR-g is a species-specific subfamily and exhibits substantial divergence from individuals in other groups [25]. Ten *P. leptostachya* sequences (PlDIR3/4/5/6/7/8/9/11/12/14) in this subfamily were of low sequence identity and unknown function (Fig. 1A).

The pattern of motif distribution across subfamilies supports the phylogenetic tree classification of PlDIRs. What we report here is the discovery of nine additional conserved motifs that are specific to some DIR clusters and allow differentiation between DIR-a, DIR-b/d, DIR-e, and DIR-g clusters, in addition to the three well-conserved motifs (motifs 1–3) that were discovered in the amino acid sequence alignments of all 15 PlDIRs. Role specificity of the motif may be connected to patterns that are conserved between individuals in the same cluster (Fig. 2).

According to Harmatha and Dinan [63], lignans exert their roles by blocking microbe-derived degradative enzymes and can act as insect antifeedants by upsetting the insect endocrine system. Lignans are a group of dimeric phenylpropanoid metabolites produced by the phenylpropanoid pathway and linked by an 8–8' bond [64]. The synthesis of phenylalanine, a building block of coniferyl alcohol, initiates this route [65, 66]. DIRs in this pathway mediate the dimerization or radial coupling of two coniferyl alcohol molecules, resulting in the synthesis of ( ±)-pinoresinol and ensuring the dimerization's stereoselectivity [20, 67]. Numerous amino acids are differentially conserved in ( +)- and (-)-pinoresinol-forming DIR proteins from various species, according to previous research. These include Gly-95, Leu-113, Phe-115, Phe-117, and Phe-130 in ( +)-pinoresinol-forming DIRs (numbering is based on PsDRR206), as well as Ala-98, Phe-116, Tyr-118, and Leu-120 (the numbering is based on AtDIR6). In this study, sequence alignment research revealed that PlDIR1 and 2 have all of the residues conserved in ( +)-pinoresinol-forming DIRs, but none of the residues linked to (-)-pinoresinol production activity (Fig. 8). Thus, these data indicate that PlDIR1/2 might be involved in ( +)-pinoresinol formation. The amino acid sequences between PlDIR1 and PlDIR2 are highly similar, with a sequence similarity of 93.6%, which indicates the same biochemical function they might share. In light of this, PlDIR1 was chosen to characterize the catalytic activity. We obtained the purified recombinant PlDIR1 protein and measured its in vitro catalytic activity with or without oxidase (Lac). As a result, in the presence of Lac, PlDIR1 could direct *E*-coniferyl alcohol coupling into ( +)-pinoresinol, effectively (Fig. 9C).

Moreover, docking analysis between PlDIR1 and bisQM, the intermediate in pinoresinol formation, was conducted to gain further insights into the relationship between protein structure and function. As a result, interaction sites and four hydrogen bonds were presented between PlDIR1 and bisQM (Fig. 11). The active site of PlDIR1 was coincident with the previously reported in ( ±)-pinoresinol formation proteins. For example, in the (-)-pinoresinol-formation protein AtDIR6, the amino acids Asp-137, Arg-144, and Thr-166 in pocket A and Tyr-106 in pocket B are vital for its catalysis function, which was proved by site-directed mutagenesis analysis [45]. In our docking analysis, four residues (Arg-143, Thr-165, Tyr-103, and Tyr-105) of PlDIR1 were found as the active sites. Particularly for Arg-143, which has the same function as previously identified proteins and also functions as a hydrogen bond donor in PlDIR1. In addition, the amino acids Asp-42 and Arg-143 that are conserved in all pinoresinol-formation proteins were also confirmed [43, 45]. These findings are consistent with the results of the PlDIR1 conserved amino acid residue alignment analysis (Fig. 8).

The expression patterns of the *DIR* genes are diverse among tissues and species [24, 27, 28, 48–50]. The expression profiles of *PlDIRs* were investigated to clarify their potential involvement in *P. leptostachya* growth and development. The majority of the *PlDIRs* displayed the highest expression in the root, followed by the leaf, stem, seed, and flower, which expressed the genes at the lowest levels of all the organs, according to the qRT-PCR analysis of the organ-specific expression (Fig. 4). These results were in line with earlier research on *B. rapa* and *I. indigotica*, whose roots showed somewhat higher expression levels [27, 28]. Moreover, it is commonly known that *DIR* genes encourage the production of lignan and that lignification takes place during normal tissue development. It's important to note that in our investigation, the root, which is the primary tissue for lignan production in *P. leptostachya*, expressed high levels of the ( +)-pinoresinol-forming gene *PlDIR1* [11]. As a result, the *PlDIRs* transcript abundance in this study in an organ-specific manner suggests that they may play roles in particular organs through the synthesis of lignans and their contribution to the developmental processes of *P. leptostachya*.

Phytohormone treatments and different conditions affect how the plant *DIR* gene is expressed. Take pepper, for instance. When this plant was subjected to SA treatments, the expression of five *CaDIRs* was induced, while three *CaDIRs* were down-regulated; when pepper was exposed to MeJA stresses, the expression of four *CaDIRs* was stimulated, while another four showed no discernible response [48]. An earlier investigation into *P. bretschneideri* revealed that MeJA and SA treatments increased the expression of PbDIR4/5/11, whereas ABA treatment up-regulated the expression of PbDIR4/5/11/19/26 [47]. A recent study also showed that, except for *PnDIR5*, *F. solani* and four signaling molecules (MeJA, SA, ETH, and H_2_O_2_) influenced *PnDIRs’* transcription [40]. In addition, at least 18 *PeDIR* genes in the ‘Nanlin 895’ poplar responded substantially to *Marssonina brunnea*, and these *PeDIRs* also showed significant responses to the application of phytohormones (ABA, MeJA, SA, ETH) [49]. In the current study, to find out the hormone response patterns of *PlDIRs*, the transcript abundance of 15 *PlDIRs* in response to MeJA, SA, and ETH treatments was analyzed in *P. leptostachya* roots and leaves (Figs. 5, 6 and 7). Overall, after treatments with three phytohormones, *PlDIR* expression levels were generally higher in leaves than in roots, which could indicate that they were primarily responsible for the biotic or abiotic stress in leaves. In detail, for *PlDIR1* and *PlDIR2*, their expressions were changed slightly or inhibited after treatments with MeJA, SA, and ETH in the roots, whereas in the leaves, they were increased by more than 60-fold and 130-fold, respectively, in response to the treatment of SA. *PlDIR3* was up-regulated after MeJA treatment in both roots and leaves. Also, its expression level was induced 23-fold by SA treatment in the root, but SA and ETH inhibited its expression in leaves. The expression of *PlDIR4*, *PlDIR5*, *PlDIR6,* and *PlDIR7* was up-regulated by SA and ETH in both roots and leaves, and they are more sensitive to SA. *PlDIR4* and *PlDIR5* in particular were upregulated more than 20,000-fold and had the strongest response to SA in leaves, indicating that they play important roles in the biotic or abiotic stress of *P. leptostachya* and deserve further investigation. In roots, MeJA inhibited the expression of *PlDIR10*, *PlDIR11,* and *PlDIR14*, as well as *PlDIR8*, *PlDIR10*, *PlDIR11*, *PlDIR14,* and *PlDIR15,* which were down-regulated by SA treatment. In addition, the expressions of *PlDIR10* and *PlDIR14* were suppressed by ETH in both roots and leaves. These findings suggest that the patterns of *PlDIRs*’ hormone response are highly intricate. According to speculation, different *PlDIR*s may participate in various signaling pathways in adverse situations. Lastly, given that *PlDIR1* was involved in lignan biosynthesis, the correlation between its expression profile and phytohormone induced lignan accumulations was evaluated. Calculated with PCCs, *PlDIR1* was connected to LA and HA biosynthesis (PCC > 0.5) after MeJA treatment and appeared to have a significant link to 6-demethoxy-LA, P-I, and P-II accumulation (PCC > 0.5 and *P* < 0.01) after SA treatment (Fig. 10), indicating that *PlDIR1* was relevant to the biosynthesis of active lignan compounds in *P. leptostachya*. However, this result needs further validation by metabolic engineering in the future.

The gene co-expression network analysis reflects the links between genes. It is a powerful method to identify genes that are expressed at the same time and pathway, which is applied in various biological studies [68]. In this study, gene expression network analysis demonstrated that Laccase and peroxidase genes were preferentially co-expressed as PlDIR partners. This allowed for the discovery of potential partner enzymes that might work with PlDIRs to construct, for instance, dimers derived from monolignol and further modify them (Fig. 12). Also, PlDIRs co-expressed with catalytic enzymes like C3H, COMT, 4CL, CcoAOMT, CCR, CAD, and CYP81Q38 may also provide some preliminary insight into the potential gene network driving the route for lignan biosynthesis. It is noteworthy that Noguchi et al. [18] indicated that PlCYP81Q38, which catalyzes ( +)-sesamin biosynthesis from ( +)-pinoresinol, was co-expressed with PlDIR1 in our study, proving the role of PlDIR1 in ( +)-pinoresinol formation. The co-expression network analysis could therefore offer more detailed and comprehensive insights into the gene functions in *P. leptostachya*.

## Conclusion

In summary, 15 *PlDIRs* were identified for the first time from the *P. leptostachya* transcription profiling database. By systematic bioinformatics analysis, four subfamilies (DIR-a, b/d, e, and g) that PlDIRs belong to were identified, and 12 motifs were well-conserved or specific in the amino acid sequence of PlDIRs proteins. PlDIR1 and 2 belong to the DIR-a subfamily, which consists of ( ±)-pinoresinol-forming DIR proteins from different species. Sequence alignment analysis revealed that PlDIR1 and 2 contain the key amino acid residues that are highly conserved in the ( +)-pinoresinol forming DIR proteins. Then, the catalytic activity of recombinant PlDIR1 protein to convert *E*-coniferyl alcohol into ( +)-pinoresinol was investigated by LC–MS/MS, and the interaction sites between PlDIR1 and substrate (bisQM) were analyzed by molecular docking, which revealed 18 active sites and 4 hydrogen bonds (Asp-42, Ala-113, Leu-138, Arg-143) in PlDIR1-bisQM complex. The increased expression in the root and leaf compared to the stem, seed, and flower seen in the organ-specific expression suggests that the root and leaf may manufacture more lignan during *P. leptostachya* development. Furthermore, MeJA, SA, and ETH significantly increased their expression, demonstrating the importance of *PlDIRs* in both biotic and abiotic stressors. This study not only clarifies the molecular and evolutionary aspects of the *PlDIR* family but also establishes the groundwork for future research on the biosynthesis of *P. leptostachya* lignan.

## Methods

### Plant materials and treatments

Seeds of *P. leptostachya* that were collected from an experimental field at the Institute of Pesticide Science, Northwest A & F University, were cultured in the chamber at 22℃/20℃, 16 h light/8 h dark with 65% relative humidity after sterilizing, rinsing, and 4 °C vernalization for 30 days. Then, each 14-day-old seedling was transplanted into an individual pot. Roots, stems, and young leaves of *P. leptostachya* six-leaf seedlings were sampled for tissue relative expression analysis. For hormone treatment, *P. leptostachya* seedlings of the six-leaf stage were uprooted from the soil and replanted in Hoagland medium, which contained 100 μM MeJA, 100 μM SA, and 1 mM ETH, respectively. The root and leaf tissues from treated *P. leptostachya* plants were harvested at 0, 6, 12, and 24 h and immediately frozen in liquid nitrogen for storage at − 80 °C for RNA extraction.

### Identification of the *PlDIRs* family

For finding the members of the *PlDIRs* family, the full-length transcriptome database of *P. leptostachya* published earlier (Accession: PRJNA551634) [11] was screened according to the annotation information. Then, candidate PlDIRs were submitted to Pfam (http://pfam.Xfam.Org/search) and SMART (http://smart.embl-heidelberg.de/) to verify whether they contained the conserved DIR domains (PF03018). The ORF for each sequence was determined using the ORF Finder available on the NCBI website (https://www.ncbi.nlm.nih.gov/orffinder). After removing the redundancy sequences, non-overlapping *PlDIRs* sequences were used for further analysis.

### Characterization analysis of PlDIR proteins

The putative signal peptide sequence of PlDIR proteins was predicted at the SignalP 4.1 server (http://www.cbs.dtu.dk/services/SignalP/), and the conserved motifs in the deduced PlDIR protein sequences were analyzed using the MEME tools (http://memesuite.org/) with default parameters [69]. Theoretical MW and pI were assessed through the ExPASy ProtParam website (http://www.expasy.org/tools/protparam.html). *N*-glycosylation sites (Asn) were searched online using the NetNGlyc 1.0 server (https://www.cbs.dtu.dk/services/NetNGlyc/). In addition, WoLF PSORT (https://wolfpsort.hgc.jp/) and CELLO v.2.5 (http://cello.life.nctu.edu.tw/) were used to predict the subcellular locations of PlDIRs [70].

### Phylogenetic analysis and multiple sequence alignment

To characterize the phylogenetic relationships between PlDIRs and DIR proteins from other plant species, MEGA 7.0 with the neighbor-joining method was used to construct a phylogenetic tree with default parameters [71]. In addition, two DIR-a proteins of PlDIRs were selected to analyze the sequence similarity with the proteins in the same subfamily by Circoletto, a web interface for comparing two sequence libraries via Circos [72]. *P. leptostachya* genes were used as the query against other *DIR* genes, and only the best match between the subject (*PlDIRs*) and query sequences was considered. Furthermore, under default settings, multiple sequence alignment was conducted with Clustal Omega (https://www.ebi.ac.uk/Tools/msa/clustalo/) and illustrated with GeneDoc software [73].

### Homology modeling and molecular docking analysis

The crystal structure of a DIR protein, PsDRR206 (C4REV.A), was used as a template to predict the theoretical model [43]. The initial homology models of PlDIR proteins were generated using the SWISS-MODEL workspace (https://www.swissmodel.expasy.org/) [74]. The interactions between intermediate 8–8′ linked *bis*-quinone methide (bisQM) in pinoresinol formation and PlDIR1 were predicted using the Discovery Studio CDOCKER software (Accelrys). The molecular structure of 8–8′ linked bisQM was generated with the use of Chem3D 19.0 and was prepared for a ligand by the operation “Apply Forcefield”. The 3D structure of PlDIR1 was prepared for the receptor protein by the operations Clean Protein, Hydrogen Add, and Apply Forcefield. The interaction or binding sites of PlDIR1 proteins were defined in previous studies [43, 45]. As a result, receptor-ligand interactions were operated by the CDOCKER protocol with the default parameters [75]. After molecular docking, the conformation with the lowest CDOCKER Interaction Energy pose was selected as the most probable binding conformation, and the types of amino acid residues and hydrogen bonds were visualized in the receptor-ligand interaction. All 3D structures of homology modeling and docking were visualized and manipulated with PyMol [76].

### Gene expression analysis

Total RNA was extracted from *P. leptostachya* tissues with the TRIzol™ Reagent (Invitrogen, USA). Then, cDNA was synthesized from 1 μg total RNA using a PrimeScriptTM RT reagent Kit with gDNA Eraser (Takara, Japan). The qRT-PCR was performed using the TB Green Premix Ex Taq™ (Tli RnaseH Plus) (Takara, Japan) with a Light Cycler 480 II system (Roche Diagnostics, Mannheim, Germany) under the following procedures: 95℃ for 30 s, 95℃ for 5 s (40 cycles), and 60℃ for 20 s. The transcript levels of the 5.8 s rRNA (GenBank Accession: DQ533822) were used as a quantitative control. All the qRT-PCR primers were designed via Primer3 (https://primer3.ut.ee/) and are listed in Additional file 6: Table S4. Each reaction was repeated with three duplications biologically and three duplications technically. The comparative threshold approach (2^−ΔΔCt^) was used to assess amplification results.

### Expression and purification of recombinant PlDIR1 protein

The ORF encoding PlDIR1 lacking a signal peptide sequence was amplified using specific primers that contain *EcoR* I and *Hind* III restriction sites in the forward and reverse directions, respectively (Additional file 6: Table S4). Then, the PCR products were inserted into the *EcoR* I/*Hind* III site of the pET29a( +) vector with the His tag using a ClonExpress II One Step Cloning Kit (Vazyme, China) to generate the pET29a( +)-PlDIR1 plasmid. The recombinant protein was expressed in *Escherichia coli* BL21 (DE3) and purified using a Ni–NTA affinity column (Qiagen, Germany). After desalting with PD-10 columns (GE, USA), the purified protein was concentrated with an Amicon® Ultra-4 centrifugal filter (Millipore, USA). A BCA protein assay kit (Epizyme, China) with bovine serum albumin (BSA) as the standard was used to measure the protein concentration. The presence of recombinant protein was confirmed by SDS-PAGE and western blot using anti-His antibodies (1:3000, CWBIO, Beijing, China) [77].

### In vitro enzyme activity assays and LC–MS/MS analysis

Enzyme activity assays were performed following Davin et al.’s method with minor modifications [20]. The total volume of standard reaction mixtures was 250 µL, which consisted of 8 mU/mL laccase from *Trametes versicolor* (Yuanye Bio-Technology Co., Ltd, China), 2 mM *E*-coniferyl alcohol, and 60 µL recombinant protein in MES-NaOH buffer (40 mM, pH 6.0). The reaction mixtures without recombinant protein or *E*-coniferyl alcohol were used as blank controls. To prepare the samples for enzyme activity reactions, the mixtures were incubated at 30 °C for 3 h, extracted twice with ethyl acetate, evaporated to dryness under a vacuum, and re-dissolved in 50% methanol. After filtering through a 0.22-μm organic membrane, samples were subjected to LC–MS/MS analysis system, with a Surveyor MS Pump Plus with Autosampler and a LTQ XL mass spectrometer (Thermo Scientific, USA) in negative ion mode. The mobile phase was 55% acetonitrile (contain 0.1% formic acid, v/v) and 45% water (contain 0.1% formic acid, v/v), under the following conditions: a flow rate of 0.3 mL/min, a Intertsil OSD-3 C18 Column (250 mm × 3.0 mm; GL Sciences Inc, Japan) at a column temperature of 35 °C and injection with 5 µL samples. Characteristic *m/z* ions were 150.5 → 151.5 for ( +)-pinoresinol.

### Lignan accumulation analysis

*P. leptostachya* root tissues (500 mg) were ground with liquid N_2_ and extracted with 5 mL of 80% methanol under sonication for 30 min. After centrifugation at 12,000 g for 10 min, the supernatant was filtered through a 0.22-µm organic membrane filter and subjected to HPLC analysis on a Nexera HPLC LC-30A system (SHIMADZU, Japan) using a 5 μm, 4.6 × 250 mm Hypersil BDS C18 column (Elite, China) with a 35 °C column temperature. A mobile phase consisting of methanol: water (70: 30, v/v) was used, with the flow rate set at 0.8 mL/min for 15 min and 10 μL for the injection volume. The UV absorbance was monitored at 280 nm. Metabolite identification and quantification was achieved as reported before [11]. The tests were run in three biological replicates, and the samples for qRT-PCR and metabolites analysis were the same.

### Gene co-expression analysis

Together with the identified *PlDIR* genes, a co-expression network was generated with the genes selected from the *P. leptostachya* transcriptome that are putatively involved in lignan biosynthesis. The complete list of these genes is presented in Additional file 4: Table S3. Gene expression data were collected from the root, leaf, and stem tissue’s full-length transcriptome database from *P. leptostachya* (Accession: PRJNA551634). Then, a gene expression correlation matrix was created using pair-wise Pearson correlation coefficients (PCC). Cytoscape 2.8.3 software was used to display a co-expression network that only included PCC values that were significant at *P* < 0.05 and had a cut-off value of 0.95 [78].

## Supplementary Information

**Additional file 1: Supplementary Table S1.** Sequence relatedness of PlDIRs.

**Additional file 2: Supplementary Table S2.** Identified motifs of PlDIR genes.

**Additional file 3: Supplementary Figure S1.** SDS-PAGE and Western Blot assay of the recombinant PlDIR1 protein.

**Additional file 4: Supplementary Table S3.** Selected co-expression genes list.

**Additional file 5: Supplementary Figure S2.** Lignan biosenthetic pathway in *P. lesptostachya*.

**Additional file 6: Supplementary Table S4.** Primers used in this study.

## Data Availability

The datasets generated and analyzed during the current study are available in the publicly accessible NCBI Sequence Read Archive (SRA) database as accession number PRJNA551634 (https://www.ncbi.nlm.nih.gov/bioproject/PRJNA551634). The nucleic acid sequences of *PlDIRs* have been deposited in the GenBank database with the following accession numbers: OQ383263 (*PlDIR1*), OQ383264 (*PlDIR2*), OQ426481 (*PlDIR3*), OQ426482 (*PlDIR4*), OQ426483 (*PlDIR5*), OQ426484 (*PlDIR6*), OQ426485 (*PlDIR7*), OQ426486 (*PlDIR8*), OQ426487 (*PlDIR9),* OQ426488 (*PlDIR10*), OQ426489 (*PlDIR11*), OQ426490 (*PlDIR12*), OQ426491 (*PlDIR13*), OQ426492 (*PlDIR14*) and OQ426493 (*PlDIR15*).

## Abbreviations

DIR: Dirigent protein
MeJA: Methyl jasmonate
SA: Salicylic acid
ETH: Ethylene
ABA: Abscisic acid
LC–MS/MS: Liquid chromatography/tandem mass spectrometry
bisQM: *bis*-Quinone methide
HA: Haedoxan A
P-I: Phrymarolins I
P-II: Phrymarolins II
ORF: Open reading frame
MW: Molecular weight
pI: Isoelectric point
chloro: Chloroplast
qRT-PCR: Quantitative real-time reverse transcription-PCR
PCC: Pearson’s correlation coefficient
LA: Leptostachyol acetate
6-demethoxy-LA: 6-Demethoxy-leptostachyol acetate
HPLC: High performance liquid chromatography
POX: Peroxidase
LAC: Laccase
C4H: Trans-cinnamate 4-monooxygenase
C3H: *p*-coumarate 3-hydroxylase
COMT: Caffeic acid 3-O-methyltransferase
4CL: 4-Coumarate-CoA ligase
CCoAOMT: Caffeoyl-CoA O-methyltransferase
CCR: Cinnamoyl-CoA reductase
CAD: Cinnamyl alcohol dehydrogenase
