# Supplementary figures and images for: Identification and functional characterization of the dirigent gene family in Phryma leptostachya and the contribution of PlDIR1 in lignan biosynthesis
Source: BMC Plant Biol. 2023 May 31;23:291. doi: 10.1186/s12870-023-04297-6 (PMC10230778; doi:10.1186/s12870-023-04297-6)

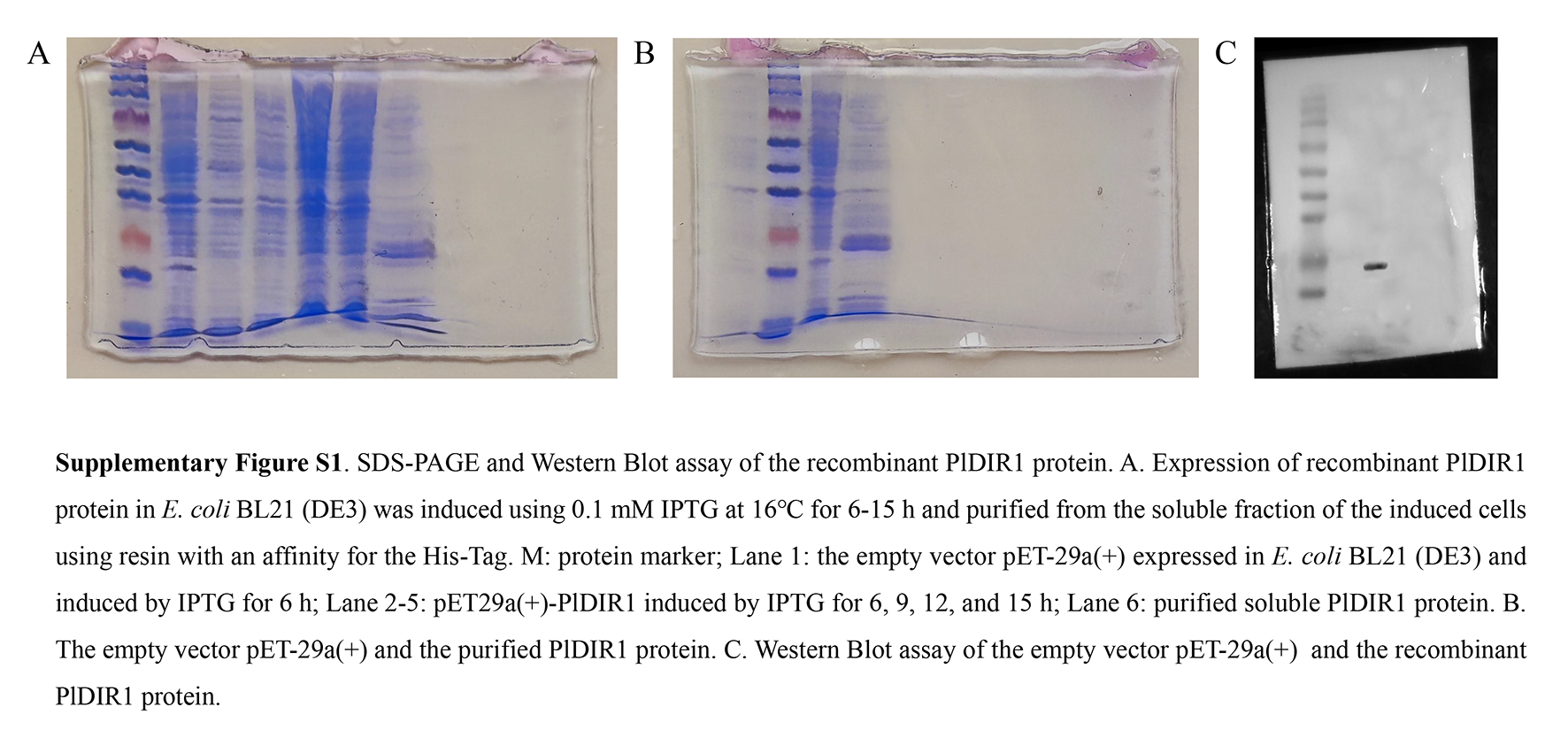

Supplement: Supplementary file 3 — Additional file 3: Supplementary Figure S1. SDS-PAGE and Western Blot assay of the recombinant PlDIR1 protein. [file 12870_2023_4297_MOESM3_ESM.tif]

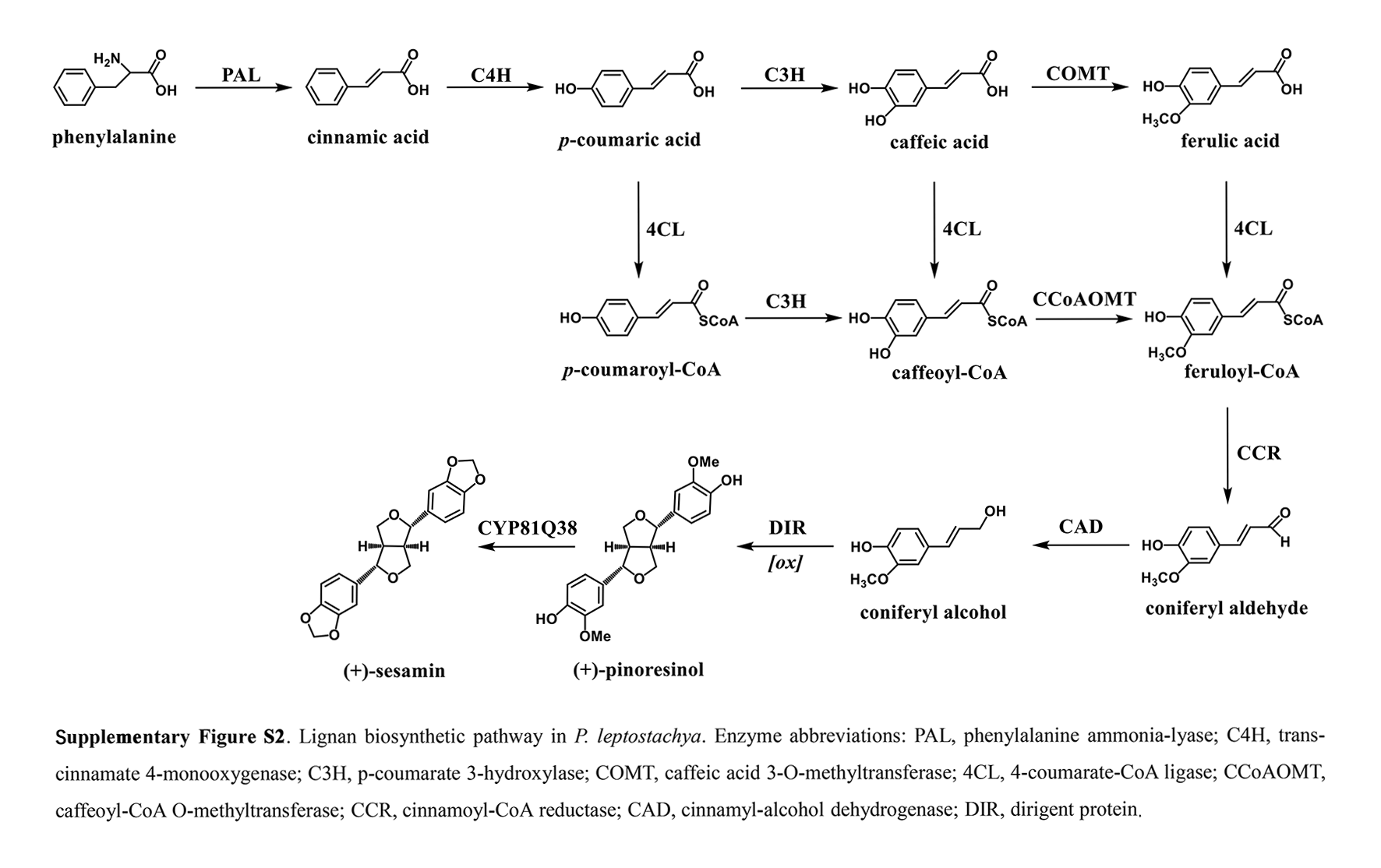

Supplement: Supplementary file 5 — Additional file 5: Supplementary Figure S2. Lignan biosenthetic pathway in P. lesptostachya. [file 12870_2023_4297_MOESM5_ESM.tif]
